# A cost-effectiveness analysis of novel stool processing methods for diagnosis of tuberculosis in children under 5 years of age in Uganda

**DOI:** 10.1186/s12913-025-13546-3

**Published:** 2025-10-13

**Authors:** Mary Gaeddert, Devan Jaganath, Hoa Thi Nguyen, Abdulkadir Civan, Pamela Nabeta, Andre Trollip, Robert Castro, Mariam Nakabuye, Moses Nsereko, Ernest Aben, Peter Wambi, Olivier Marcy, Eric Wobudeya, Adithya Cattamanchi, Alfred Andama, Manuela De Allegri, Claudia M. Denkinger

**Affiliations:** 1 Division of Infectious Disease and Tropical Medicine, University Hospital Heidelberg, Im Neuenheimer Feld 324, 69120 Heidelberg, Germany; 2 Division of Pediatric Infectious Diseases, University of California San Francisco, San Francisco, USA; 3 Center for Tuberculosis, Institute of Global Health Sciences, University of California San Francisco, San Francisco, USA; 4 Heidelberg Institute of Global Health, Heidelberg University Hospital and Medical Faculty, Heidelberg University, Heidelberg, Germany; 5 FIND, Geneva, Switzerland; 6 Uganda Tuberculosis Implementation Research Consortium, Walimu, Kampala Uganda; 7 National Institute for Health and Medical Research (Inserm), UMR 1219, Research Institute for Sustainable Development (IRD), University of Bordeaux, EMR 271, Bordeaux, France; 8 Division of Pulmonary Diseases and Critical Care Medicine, University of California Irvine, Irvine, USA; 9 Makerere University College of Health Sciences, Kampala, Uganda; 10 German Center of Infection Research (DZIF), Partner Site, Heidelberg, Germany

**Keywords:** Tuberculosis, Pediatric, Diagnostics, Cost-effectiveness, Stool

## Abstract

**Background:**

Stool-based molecular assays for childhood tuberculosis (TB) diagnosis have shown promise as an alternative to respiratory sample testing. While implementation is underway, evidence on cost-effectiveness is needed. Therefore, we aimed to evaluate the costs of stool testing with Xpert Ultra and model the cost-effectiveness of implementation scenarios at lower levels of care.

**Methods:**

We measured costs for three new stool processing methods: Simple One-Step (SOS), Stool Processing Kit, and Optimized Sucrose Flotation. Each method mixed stool with Xpert Sample Reagent buffer, incubated to allow sedimentation, and then dispensed the supernatant into the Xpert Ultra cartridge. While the other methods required additional buffer and supplies, SOS used only the Sample Reagent. Using the least costly method, we modeled implementation for children under 5 years with presumptive TB at primary health clinics or district hospitals in Uganda. Clinical diagnosis with treatment-decision algorithms was compared to stool testing at primary clinics, stool testing at primary clinics with referral to district hospitals if negative, or evaluation only at district hospitals with Xpert Ultra testing on respiratory samples. Using decision-tree models, we calculated the cost in international dollars (I$) per life-years saved (LYS) and the incremental cost-effectiveness ratio (ICER) assessed against the country-specific willingness to pay threshold. One-way and probabilistic sensitivity analyses were conducted.

**Results:**

SOS was the least costly stool processing method. Compared to diagnosis with only treatment-decision algorithms, the ICER of SOS/Ultra at primary clinics was I$1041.71/LYS, SOS/Ultra with referral was I$874.82/LYS, while the district hospital strategy was dominated. Sensitivity analyses showed stool testing was cost-effective compared to only clinical diagnosis if TB prevalence at primary clinics was above 5.7%, with higher diagnostic accuracy of stool-based testing, or lower testing costs.

**Conclusions:**

For young children, stool testing at primary clinics, with or without referral to district hospitals, lowered costs in relation to lives saved compared to implementing at district hospitals alone or only clinical diagnosis using the treatment-decision algorithms.

**Supplementary Information:**

The online version contains supplementary material available at 10.1186/s12913-025-13546-3.

## Background

Of the estimated 1.3 million children with tuberculosis (TB) each year, about half are not reported to public health programs [1]. This gap is in large part due to missed diagnoses that cause delays in treatment and increase morbidity and mortality. Young children present unique diagnostic challenges because they often show non-specific symptoms and cannot expectorate sputum for microbiologic testing [2, 3]. To address these barriers, non-sputum-based diagnostics, which can be implemented at or near the point of care at peripheral health facilities, are urgently needed.

Stool has been a promising sample type for molecular TB testing, and the World Health Organization (WHO) has endorsed its use [4]. To facilitate implementation in peripheral facilities, three centrifuge-free stool processing methods have been developed for use with Xpert Ultra MTB/RIF (Cepheid, USA): the Stool Processing Kit (SPK, FIND), Simple One-Step (SOS, KNCV), and Optimized Sucrose Flotation (OSF, TB Speed). Although each of these methods involves different levels of complexity and materials, a multi-center study has demonstrated that these methods perform similarly and are acceptable and usable for laboratory staff [5]. However, there are several ongoing questions that limit implementation of stool Xpert Ultra. First, the costs of each processing method have not been compared. Second, although stool testing could be integrated into the WHO treatment decision algorithms (TDAs) for childhood TB in primary health centers (PHCs) [6], their cost-effectiveness compared to the symptom-based scoring system alone is unknown. Third, because Xpert Ultra on respiratory samples has higher sensitivity than stool Xpert Ultra, it is unclear if referral to a higher-level facility for respiratory testing is more cost-effective.

In order to address these questions and inform implementation strategies, we calculated the costs of each stool processing method and modelled the potential cost-effectiveness of different scenarios for implementing stool testing at peripheral level outpatient clinics. Specifically, we projected the cost-effectiveness of implementing stool Xpert at PHCs compared to clinical diagnosis with the treatment decision algorithm alone. Furthermore, we estimated the cost-effectiveness of diagnosis at PHCs (stool and TDA) compared to referral for diagnosis at district hospitals (DH) only, or a mixed strategy which includes both evaluation at PHCs and referral to DH.

## Methods

### Micro-costing study

We conducted a micro-costing study to calculate the costs of each stool processing method. Details of the stool processing methods are described elsewhere, including the materials and time needed to conduct each method [5]. Briefly, all methods followed a similar approach of mixing the stool sample with buffer. SPK had a pre-assembled kit which included a squeeze bottle with mixing beads and a filter cap, and its own buffer for stool processing. OSF required both a sucrose solution, which had to be made in batches every month, and the Xpert Sample Reagent buffer. SOS involved simply adding the stool directly to the Xpert Sample Reagent bottle. After mixing, all methods had an incubation step to allow sedimentation and then transferred the supernatant into the Xpert Ultra cartridge.

Since these differences in processing methods impact cost, we used a bottom-up micro-costing approach following three sequential steps: identification of resources for laboratory processes, measurement of resource consumption, and valuation of resource consumption. Given that the three methods require similar infrastructure (buildings and electricity) and equipment, we included only recurrent costs (staff time, reagents, and consumables) related to conducting stool testing. Because laboratory staff time represents a major cost, we conducted a time and motion study to record the exact time that technicians spent processing the stool for each method. The time for incubation and sedimentation steps were specified in the protocol for each method, and actual procedure times were recorded for stool samples processed. The time for preparing the sucrose solution for OSF was also recorded. The time for Xpert Ultra testing was not included in the time and motion study, as it was the same for all methods. In clinical practice, an invalid or error result would be repeated, and require additional time and materials. The cost of invalid-repeat testing was thus calculated as a function of the invalid rate and cost per repeat test. The most cost-effective stool processing method was used in the implementation models.

### Model structure and comparisons

The study population comprised children under 5 years of age with symptoms of presumptive TB being evaluated for pulmonary TB. The setting used for the analyses were outpatient clinics in PHCs and DHs in Uganda, one of the sites of the multi-center stool diagnostic accuracy study [5]. In Uganda, most resources for diagnostic testing, including X-ray facilities, laboratories with GeneXpert, and clinical resources to perform gastric aspirates are located at centralized facilities such as district hospitals. Therefore, they often perform Xpert Ultra testing on samples received from primary health centers, including sputum and stool.

**Fig. 1:**
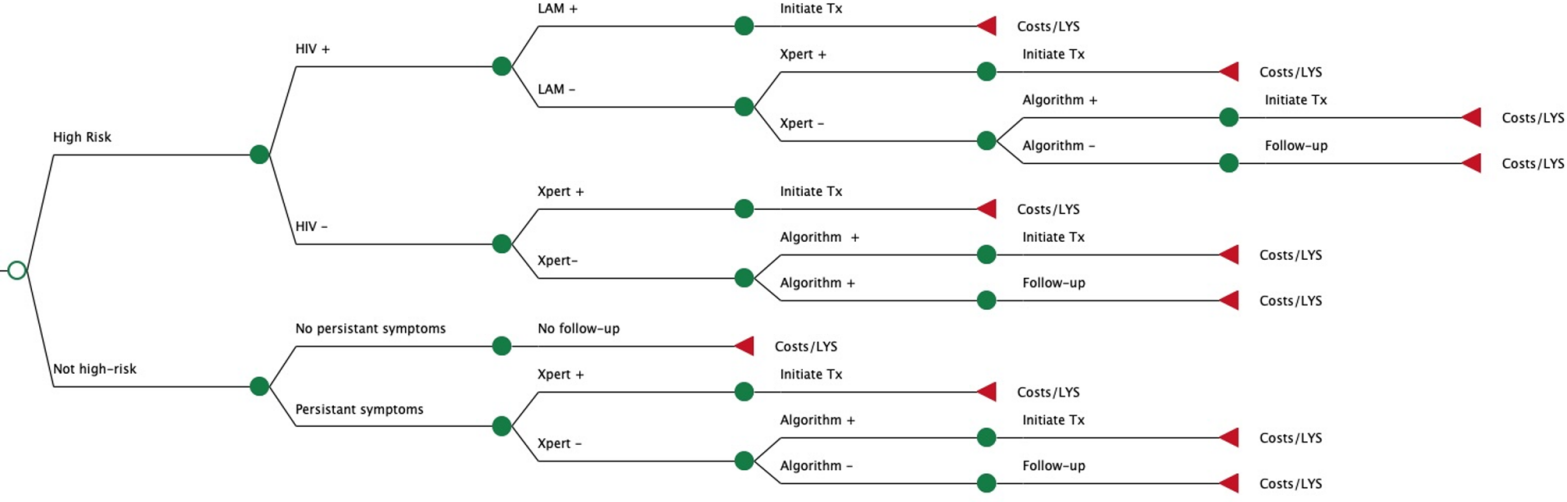
Structure of decision tree models. Legend: This figure shows a simplified version of the clinical pathway for each model. The sample for Xpert Ultra testing (stool or gastric aspirate) and treatment decision algorithm used (TDA-A or B) vary according to the strategy

We developed decision-tree models following the clinical pathway from initial evaluation to an outcome of survival or death based on the recommendations for the treatment-decision algorithms [6] (Fig. 1, Additional File 1). Children at high risk for rapid disease progression (under 2 years of age, with HIV, or with severe acute malnutrition) were tested for TB immediately with Ultra if a sample was available. Children with HIV also had urine collected for lipoarabinomannan (LAM) testing. The children not in a high-risk group returned for a follow-up visit after two weeks and those with persistent symptoms continued for additional evaluation, otherwise there were no further steps. If a urine or stool sample was not available, the child would move to the next step. While collecting these samples is non-invasive, practice has shown that children cannot always provide a sample during the clinic visit [5]. If a microbiologic diagnosis with urine LAM or Xpert Ultra is not reached, the last step is clinical evaluation with the treatment-decision algorithms. These use a scoring system to guide clinicians in reaching a diagnosis; i.e. if the score is above a set threshold then TB treatment should be initiated [6]. There are two versions of the algorithms: the scoring system for TDA-A includes chest X-ray (CXR) findings for settings where X-ray is available, and TDA-B includes only clinical signs and symptoms. Once a final diagnosis of TB is reached, the child is initiated on treatment and the outcomes are classified as survival, death from TB on treatment, or death from other causes. If a TB diagnosis was missed or if a child was lost to follow-up during treatment, outcomes included death from TB with no or partial treatment, self-cure, or death from other causes.

Four different strategies were compared. The standard of care at PHCs included only clinical diagnosis with the TDA-B and no Xpert Ultra testing (‘TDA-B’ strategy). With stool testing at PHC (‘Stool’ strategy), a stool sample was collected and transported to the DH laboratory for processing and Xpert Ultra testing. If the stool was positive, the child was initiated on TB treatment. If the stool was negative, or a sample was not collected, clinical diagnosis was made using TDA-B. For the third strategy, all children were evaluated at a district hospital (‘DH’ strategy), and had a gastric aspirate collected for Xpert Ultra. If negative, clinical diagnosis was performed with the TDA-A, incorporating CXR findings. The final strategy started with stool testing at PHC, and if the stool was negative or not collected, a portion of children would be referred to district hospital for evaluation (‘Stool-Referral’).

The estimates of diagnostic accuracy for each stool processing method were taken from the study by Jaganath et al. [5], which included children in Uganda, a majority of whom were younger than 5, and was the site of the micro-costing study. Additional clinical and cost parameters were taken from the literature (Table 1). Several clinical parameter estimates were obtained from TB-Speed’s multi-site study on decentralization of TB testing [7] and the authors (OM, EW) provided data for PHCs and DHs at the Ugandan site. Due to limited data available on children at PHC level, we also consulted expert opinion (see acknowledgement) for input on clinical parameters with the most uncertainty, including TB prevalence, stool sample collection, and resolution of symptoms after follow-up. Due to limited data available on pre-diagnostic loss and how implementation of new diagnostic strategies may impact the pathway, loss to follow-up before or during the diagnostic process was not included.

**Table 1:** The model parameter estimates for children with presumptive TB under 5 years of age

Clinical Parameters	Base Case	Range	Distribution	Reference
Prevalence of TB, PHC	0.03	0.02–0.04	Beta	[7]
Prevalence of TB, DH	0.10	0.08–0.12	Beta	[7]
Prevalence of HIV, PHC	0.05	0.04–0.06	Beta	[8]
Prevalence of HIV, DH	0.10	0.08–0.12	Beta	[8]
Proportion high-risk, PHC	0.60	0.48–0.72	Beta	[7]
Proportion high-risk, DH	0.70	0.56–0.84	Beta	[7]
Stool sample available	0.65	0.52–0.78	Beta	[7]
Urine sample available	0.65	0.52–0.78	Beta	[9]
Persistent symptoms, TB positive	0.80	0.64–0.96	Beta	[10, 11]
Persistent symptoms, TB negative	0.20	0.16–0.24	Beta	[10, 11]
Sensitivity of respiratory Xpert Ultra, in children without HIV	0.73	0.65–0.80	Beta	[12–14]
Specificity of respiratory Xpert Ultra, in children without HIV	0.97	0.96–0.98	Beta	[12–14]
Sensitivity of respiratory Xpert Ultra, in children with HIV	0.64	0.44–0.80	Beta	[12–14]
Specificity of respiratory Xpert Ultra, in children with HIV	0.98	0.93-1.0	Beta	[12–14]
Sensitivity of SPK	0.37	0.29–0.46	Beta	[5]
Specificity of SPK	0.98	0.96–0.99	Beta	[5]
Sensitivity of SOS	0.39	0.27–0.51	Beta	[5]
Specificity of SOS	0.97	0.94–0.99	Beta	[5]
Sensitivity of OSF	0.31	0.20–0.44	Beta	[5]
Specificity of OSF	0.97	0.93–0.99	Beta	[5]
Sensitivity of urine LAM	0.42	0.15–0.75	Beta	[15]
Specificity of urine LAM	0.81	0.75–0.86	Beta	[15]
Referral from PHC to DH, high risk	0.35	0.28–0.42	Beta	[16]
Referral from PHC to DH, low risk	0.20	0.16–0.24	Beta	[7]
Sensitivity of TDA-B	0.86	0.68–0.94	Beta	[17]
Specificity of TDA-B	0.30	0.13–0.56	Beta	[17]
Sensitivity of TDA-A with CXR	0.88	0.71–0.95	Beta	[17]
Specificity of TDA-A with CXR	0.37	0.15–0.67	Beta	[17]
Complete TB treatment	0.70	0.56–0.84	Beta	[18, 19]
TB mortality, on treatment	0.03	0.007–0.07	Beta	[20]
TB mortality, incomplete treatment	0.17	0.12–0.23	Beta	[20]
TB mortality, untreated	0.44	0.37–0.5	Beta	[20]
Self-cure	0.30	0.18–0.42	Beta	[21]
Lost to follow-up	0.20	0.12–0.28	Beta	[10, 19]
Mortality, non-TB	0.013	0.01–0.02	Beta	[22]
Life expectancy, Uganda	66.7		Beta	[22]
**Cost Parameters**,** I$**	**Base Case**	**Range**	**Distribution**	**Reference**
Outpatient visit, PHC	$3.17	2.54–3.81	Gamma	[23]
Outpatient visit, DH	$4.46	3.57–5.35	Gamma	[23]
HIV testing	$8.89	7.11–10.67	Gamma	[24]
Urine LAM testing	$4.79	3.83–5.75	Gamma	[25]
Chest X-ray	$11.11	8.89–13.34	Gamma	[26]
Stool collection	$1.94	1.55–2.33	Gamma	[27]
Gastric aspirate	$5.09	4.07–6.11	Gamma	[27]
Sample transport	$1.59	1.39–1.79	Gamma	[28]
Xpert Ultra testing	$23.12	18.50-27.74	Gamma	[28, 29]
Cost of TB treatment, 6 months	$354.31	238.16-516.02	Gamma	[30]

The analysis adopted a health system perspective, using a time horizon of one year for program implementation and costs of the entire pathway including treatment. Patient costs, such as transportation, were not included. Costs incurred in different years were converted to 2023 International dollars (I$) using World Bank inflation data [31, 32]. Health outcomes were calculated as life year saved (LYS) over the child’s lifetime with a discount rate of 3% [33]. The incremental cost-effectiveness ratio (ICER) per LYS was calculated by dividing the incremental costs by the incremental effects. When diagnosed with TB and initiated on treatment, children were assumed to live their full life expectancy, and death from TB would be averted. Results were appraised in reference to the maximum willingness to pay (WTP), calculated using a country-specific cost effectiveness threshold [34] based on the 2023 per capita GDP for Uganda [35]. We extrapolated Uganda’s WTP in 2023 from its estimated PPP-adjusted threshold in 2013, resulting in a WTP threshold of I$569. One-way and probabilistic sensitivity analyses were conducted to evaluate how the range of uncertainty in model parameters impacted the ICER estimates. TreeAge Pro 2024 was used for analysis and the model is included in Additional File 1. The study was approved by the Heidelberg University Ethics Committee and reported following the Consolidated Health Economic Evaluation Reporting Standards (CHEERS) 2022 guidance (Additional File 2) [36].

## Results

### Micro-costing study

The time and motion results were recorded for 51 stool samples and four batches of sucrose solution processed between October 2020 to February 2021 (Additional file 3). The median procedure time was the longest for OSF, including time to prepare the sucrose solution, which resulted in the highest cost for staff time (Table 2). SOS had the lowest cost of the three stool processing methods at $12.50 per test, followed by SPK at $19.13 and OSF at $19.51. The higher rate of invalid Xpert Ultra results for the OSF method required more repeat testing and increased the procedure cost. The cost of consumables was the lowest for SOS as very few materials are required besides those provided with the Xpert Ultra cartridge. From the diagnostic accuracy study, SOS had the highest sensitivity (38.6%), compared to SPK (36.9%) and OSF (31.3%), although the differences were not statistically significant. Therefore, SOS has the lowest cost and highest effectiveness and was used in the implementation models.

**Table 2:** Results of the micro-costing study for each stool processing method

Item	Simple One-Step (SOS)	Stool Processing Kit (SPK)	Optimized Sucrose Flotation (OSF)
Time and motion results (in minutes)			
Incubation/sedimentation time	10/10	30*	30/15
Procedure time, median (range)	23 (21–30)	23 (18–40)	43 (32–74)
Sucrose preparation time, per aliquot	N/A	N/A	2
Non-determinate results [5]	11.3%	9.7%	12.6%
Costs, 2023 I$			
Staff time (range)	$2.31 (2.02–2.90)	$2.31 (1.73–3.85)	$4.27 (3.08–7.13)
Consumables	$0.95	$7.16	$5.09
Xpert MTB/RIF Ultra [37]	$7.97	$7.97	$7.97
Cost of invalid-repeat testing	$1.27	$1.69	$2.18
**Total cost**,** range**	$12.50 (12.22–13.09)	$19.13 (18.56–20.67)	$19.51 (18.33–22.37)

*The SPK method does not have a sedimentation step

### Comparison of implementation strategies

**Fig. 2:**
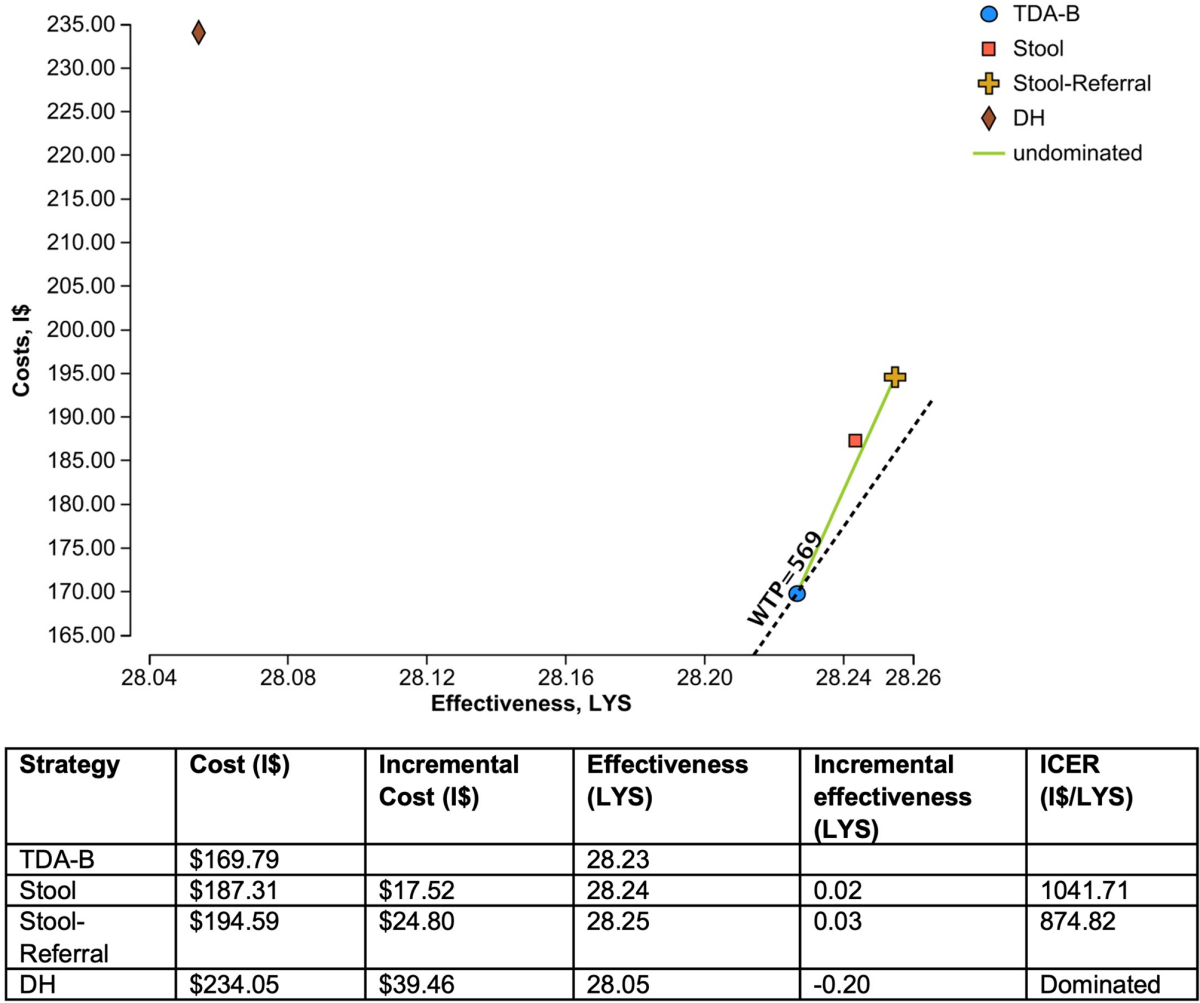
Cost-effectiveness analysis results. Legend: LYS = life-years saved, I$ = International dollars, dotted line = Willingness to pay (WTP) threshold of $569

The TDA-B strategy had the lowest cost at I$169.79, and the others had proportionately higher costs as more diagnostics were included (Fig. 2). The effectiveness was very similar for the TDA-B (28.23 LYS), Stool (28.24 LYS), and Stool-Referral strategies (28.25 LYS) as they all began with the same population at PHCs and the additional testing resulted in only small gains in incremental effectiveness. The Stool strategy had both a greater effectiveness and higher cost than diagnosis with only TDA-B at primary health clinics, but with an ICER of I$1,042/LYS was above the WTP threshold of I$569/LYS. The Stool-Referral strategy also had higher effectiveness and costs due to the increased proportion with microbiologic confirmation from Xpert Ultra on respiratory samples, with an ICER of $874.82/LYS compared to TDA-B alone. Evaluating children only at district hospitals had both the highest cost and was the least effective compared to other strategies, so the strategy was dominated. The additional diagnostic work-up detected more TB cases and was more costly. However, the higher prevalence of TB in this population resulted in more missed cases and a lower effectiveness overall.

### Sensitivity analyses

**Fig. 3:**
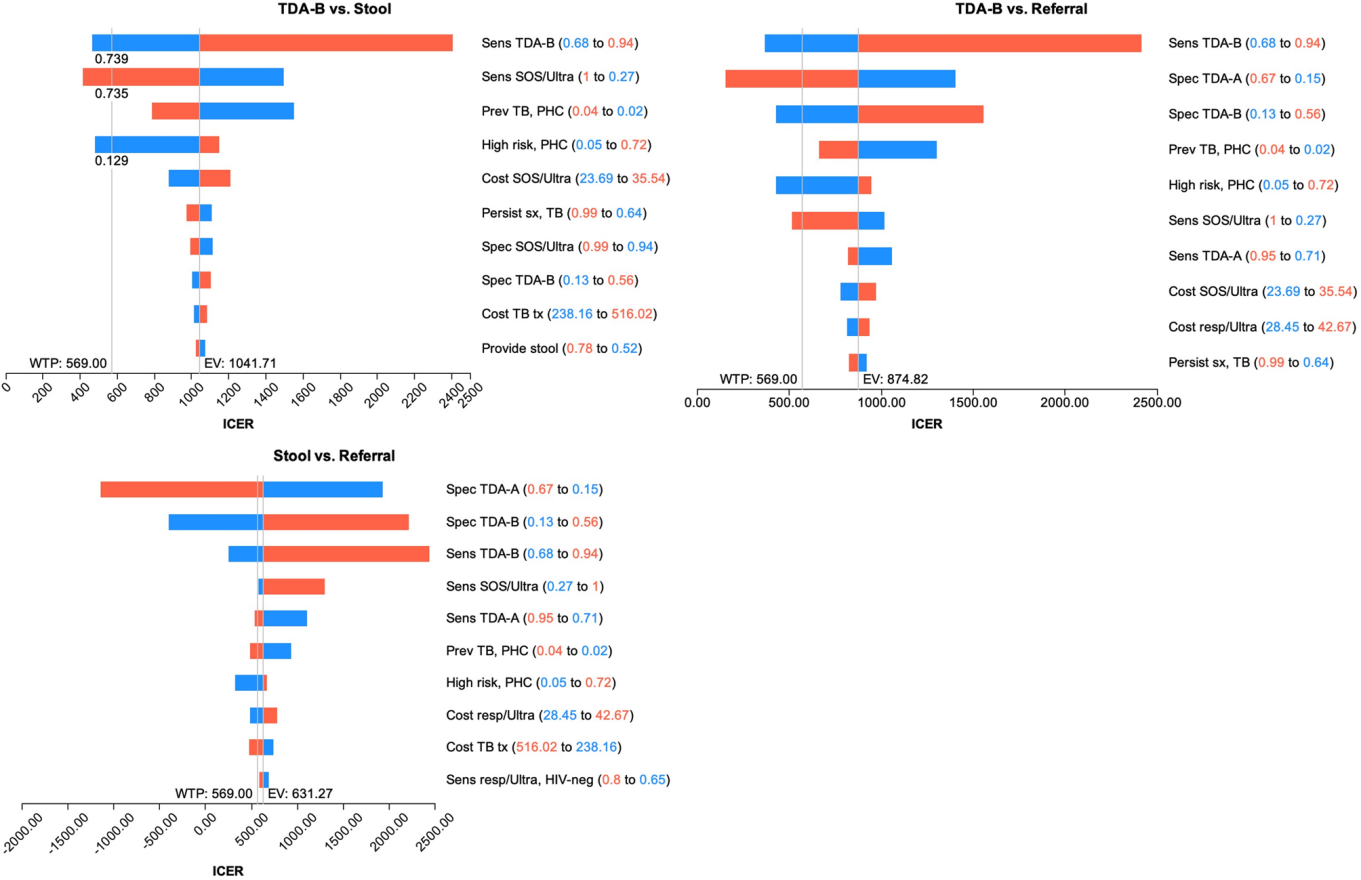
One-way sensitivity analyses of all parameters. Legend: WTP=willingness to pay threshold; EV=expected value, blue=low range of parameter, red=high range of parameter. Variables are shown in order of decreasing impact

One-way sensitivity analyses explored how the uncertainty of individual parameters impacted cost-effectiveness of different scenarios. The sensitivity and specificity of the TDA’s, prevalence of TB disease, sensitivity of SOS/Ultra, cost of SOS/Ultra, and proportion of children in a high-risk group were the main drivers across all strategies (Fig. 3). The main differences between the Stool and Stool-Referral strategies were related to the performance of the TDAs. The Stool-Referral strategy would be cost-effective compared to both TDA-B and stool testing if the specificity of TDA-A was higher, or the sensitivity and specificity of TDA-B was lower.

**Fig. 4:**
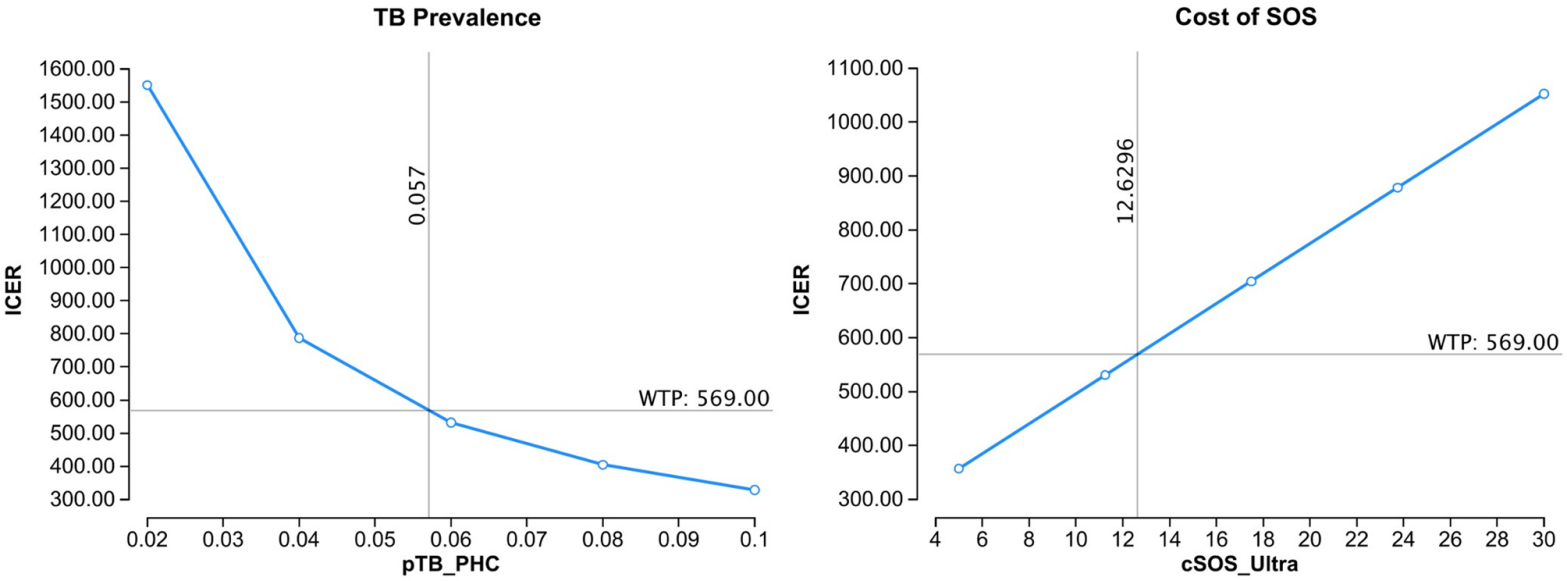
One-way sensitivity analyses for individual parameters of SOS compared to TDA-B at primary health centers. Legend: horizontal line = Willingness to Pay threshold, vertical line = threshold value for cost-effectiveness

Stool testing at PHC would be cost-effective compared to TDA-B alone if the prevalence of TB disease were above 5.7% (vs. 3.0% in model), or if the cost of SOS/Ultra decreased from $29.62 to below $12.63 (Fig. 4). Furthermore, as the sensitivity of stool in young children is low, an increase in the sensitivity of SOS/Ultra by at least 30% to above 73.5% would be needed. Other changes in parameters that would make the Stool strategy cost-effective would be if the TDA-B sensitivity was lowered to < 73.9%, or if the proportion of children in the high-risk group was lowered from 60% to 12.9%. For children not in a high-risk group, the additional follow-up visit reduces costs because many children without TB will experience resolution of symptoms and not proceed for further testing. However, the proportion of children in the high-risk group varies by setting, and the risk of increased morbidity and mortality from delayed diagnosis or loss to follow-up may outweigh the reduced cost of testing.

**Fig. 5:**
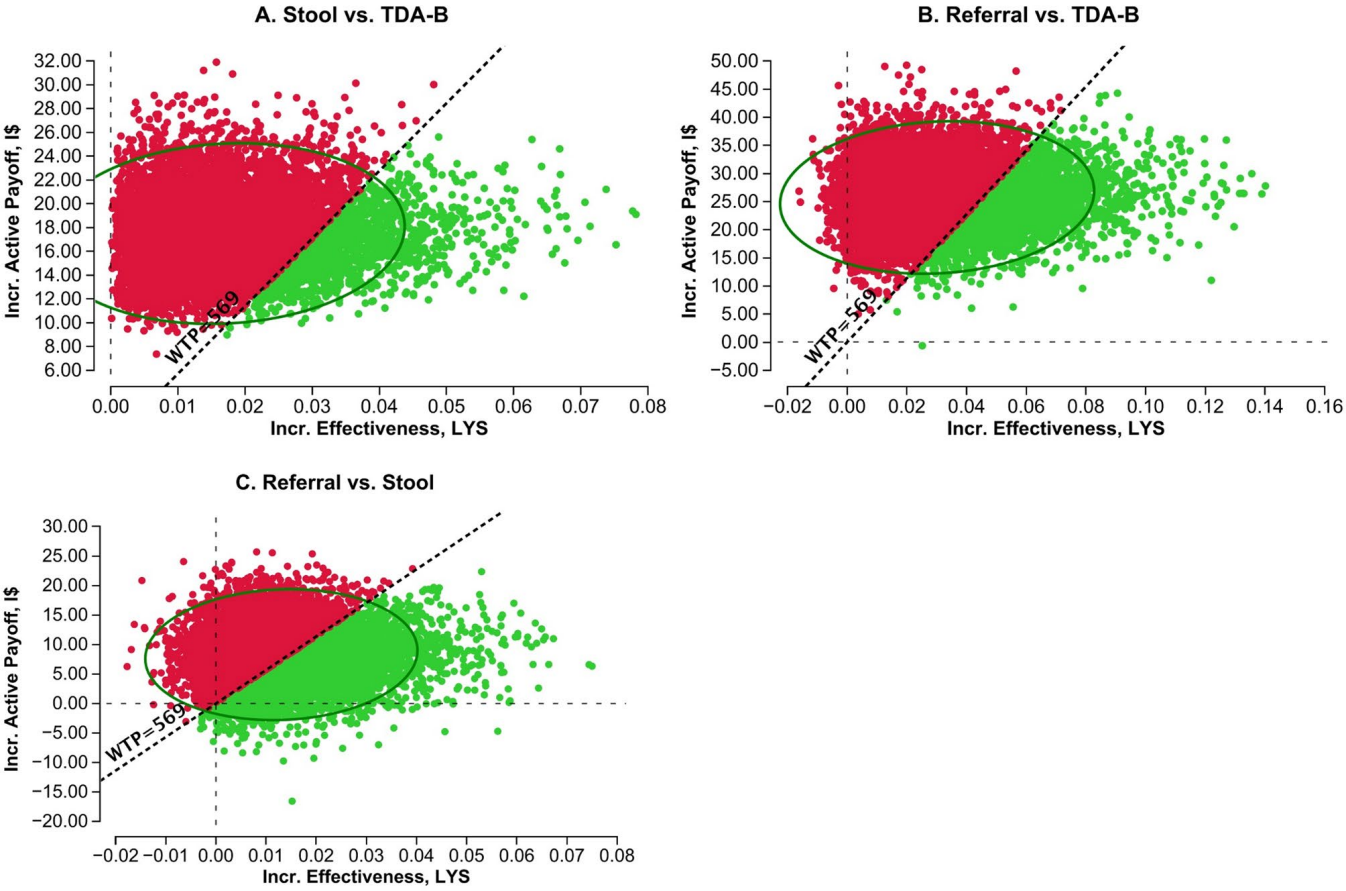
Incremental cost-effectiveness scatterplots. Legend: Incremental Cost-Effective scatterplot using Monte-Carlo simulation with 10,000 iterations, circle = 95% confidence region, dotted line = WTP (willingness to pay threshold), green = strategy below WTP, red = strategy above WTP. **A**: Comparator = Stool, Baseline = TDA-B. In 12.4% of the iterations, Stool was < WTP (green); in 87.6% Stool was > WTP (red). **B**: Comparator = Referral, Baseline = TDA-B. In 23.1% of the iterations, the Referral strategy was < WTP (green); in 76.9% the Referral strategy was > WTP (red). **C**: Comparator = Referral, Baseline = Stool. In 42.2% of the iterations, the Referral strategy was < WTP (green); in 57.8% the Referral strategy was > WTP (red)

**Fig. 6:**
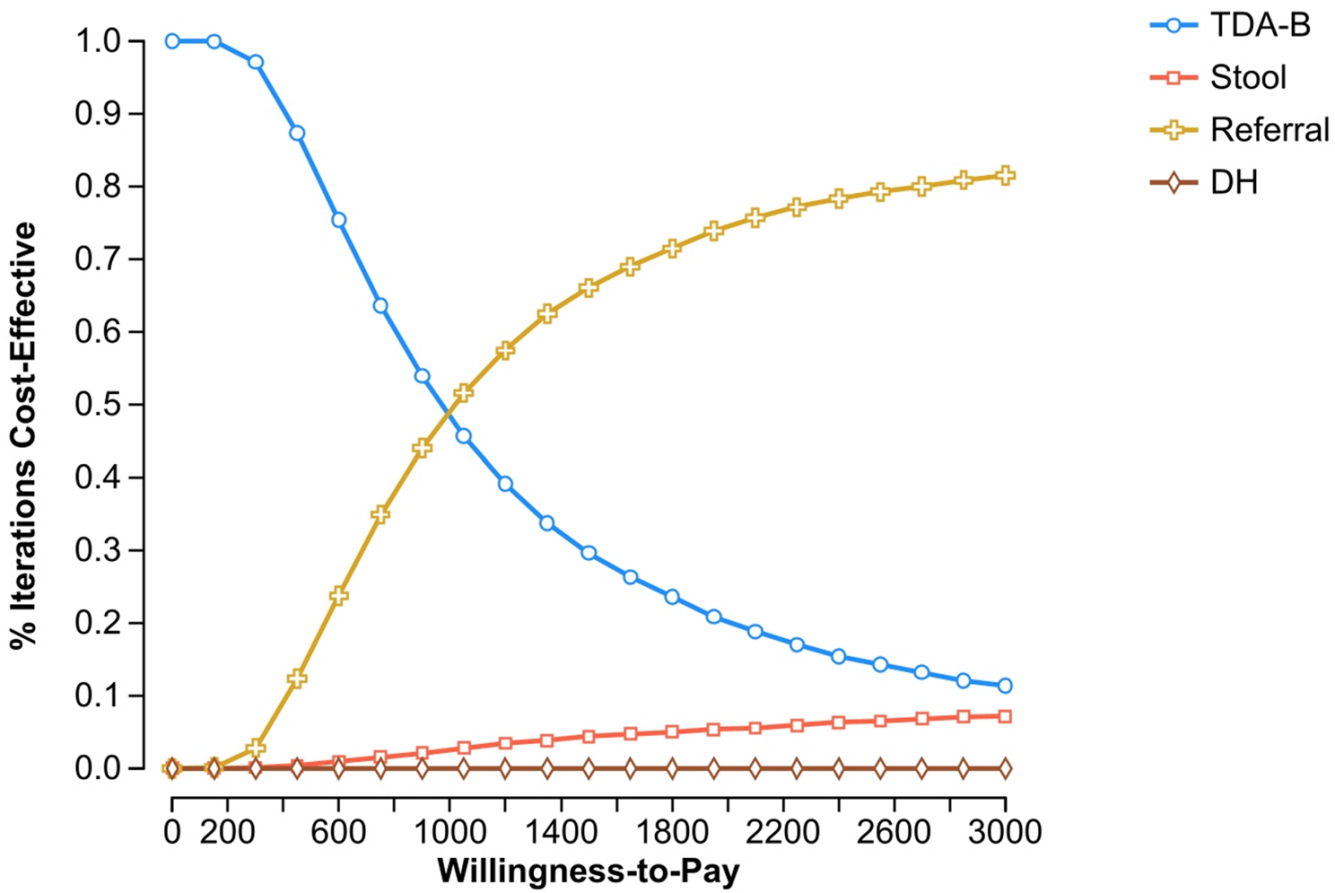
Cost-effectiveness acceptability curve. Legend: Probabilistic sensitivity analysis using Monte-Carlo simulation with 10,000 iterations; separate lines for each strategy showing the probability each strategy is cost-effective at a given WTP threshold

In probabilistic sensitivity analyses, incremental cost-effectiveness scatterplots for the Stool and Stool-Referral strategies compared to TDA-B showed that most values are clustered in the first quadrant, indicating that these strategies are more effective than TDA-B alone but more costly (Fig. 5). However, both strategies exceed the WTP threshold in the majority of iterations. When the Stool-Referral strategy is compared to the Stool strategy, the values are also clustered in the first quadrant but distributed across the other quadrants and one-third of iterations would be cost-effective. The acceptability curve shows that, as the WTP threshold increases, the probability of the Stool-Referral strategy being cost-effective compared to the other strategies increases (Fig. 6). At a WTP threshold of I$1,000 the Stool-Referral strategy, with stool and respiratory testing, is more cost-effective than TDA-B alone. Both the Stool and DH strategies remain at a low probability of cost-effectiveness even with a high WTP threshold.

## Discussion

Stool-based TB testing can increase access to diagnostics for children, but there is limited data on the cost-effectiveness of different implementation strategies. We found that of the current stool processing methods, SOS was the least costly. For children under 5 in Uganda, SOS/Ultra testing at a primary health center, for all children or with partial referral, was more cost-effective than evaluating all children at district hospitals with respiratory sampling and CXR. However, none of the implementation strategies proved to be more cost-effective than clinical diagnosis with TDA-B alone, and estimates were most impacted by TB prevalence, cost and sensitivity of SOS/Ultra, and diagnostic accuracy of the treatment-decision algorithms. These findings suggest that stool-based TB testing at primary health centers has better value than evaluation at district hospitals, but that further improvements in diagnostic accuracy and consideration of the appropriate setting are needed to improve cost-effectiveness.

The SOS method was the least costly of the three processing methods compared to OSF and SPK. This was due to the low cost of consumables as it does not require supplies other than those included with Xpert. The low cost per test also resulted in a low cost for repeat testing in case of invalid results. Due to the low cost and relative ease of use, the SOS method has been recommended for implementation [38, 39].

In this analysis, the stool strategy was not cost-effective compared to diagnosis with only treatment decision algorithm at primary health clinics. The clinical scoring of the algorithm can be performed during a routine outpatient visit and has higher sensitivity than stool testing. A recent modelling analysis using a similar approach found that stool testing for children was cost-effective in Indonesia and Ethiopia [40]. However, their approach estimated a higher prevalence of disease, a higher sensitivity of stool testing, and used estimates for clinical diagnosis with lower sensitivity and higher specificity than the newly recommended TDAs used in our models.

Sensitivity analyses showed that TB prevalence has a significant impact on the cost-effectiveness. There is limited data available on the TB prevalence at the PHC level as most studies on childhood TB are done in referral hospitals. If the true prevalence of TB disease in children presenting to primary health clinics is higher than we modelled, then stool testing would be more cost-effective. However, we are confident that prevalence estimates from the TB Speed study, which enrolled a population similar to that modelled, are reliable.

The sensitivity and cost of SOS/Ultra testing also influenced the cost-effectiveness. Other recently published studies have reported higher sensitivities of SOS/Ultra, ranging from 60 to 91% [38, 41] although several of these were early-stage studies with a larger proportion of older children who were hospitalized. If SOS/Ultra performs better than our estimates, stool testing would be more cost-effective. However, since stool testing is mainly aimed for implementation at PHCs, where children are more likely to have early-stage paucibacillary disease, the sensitivity may be lower. Also, the sensitivity of SOS/Ultra was about 10% lower in the under 5 age group of the diagnostic accuracy study [5]. We did not use these estimates in our model due to the small sample size but results with the overall estimate may be optimistic for children under 5. Regarding the cost of stool testing, other low-complexity PCR tests, such as Molbio Truenat, are currently used for sputum and can be performed at point-of-care [42]. While the negotiated prices are similar to Xpert [29] and the Molbio assay would need to be validated for stool samples, testing at point-of-care would eliminate the expense and delays of sample transport, reducing the total cost per test.

Each strategy includes a final step of clinical diagnosis with the TDA’s. These were developed using data mainly from children at referral hospitals and currently have a provisional recommendation. As the TDAs are evaluated in populations at PHC level with less severe disease presentation and lower prevalence, the estimates of diagnostic accuracy are likely to decrease, making stool testing more effective in comparison. For the stool testing to be cost-effective, a decrease in the TDA’s accuracy of at least 10–15% would be required if all other parameters were unchanged.

The Stool-Referral strategy had a higher cost and was more effective at detecting TB cases than the stool only strategy due to the addition of respiratory Xpert testing and the algorithm with CXR. Both stool strategies at PHC were more cost-effective than district hospital strategy. Centralized testing is the current standard of care in most countries because there is no capacity at PHCs to collect respiratory samples from young children or expertise to make a clinical diagnosis.

Also, there are additional benefits of bacteriological confirmation with Xpert Ultra not reflected in these models. In settings with a higher prevalence of drug resistance, the detection of rifampicin resistance and the ensuing appropriate treatment would likely result in better outcomes. The use of stool testing at PHC may also allow for the earlier detection and treatment, preventing progression to more severe disease.

Our findings comparing different strategies will be useful to guide implementation of stool testing. Despite the limited cost-effectiveness, the added value of stool testing is considered highly in some settings and implementation is already underway in some countries. It will be important to update the cost-effectiveness models with country-specific parameters as additional data becomes available.

### Strengths and limitations

This analysis had several limitations. First, there is limited data available for childhood TB, especially for populations presenting at primary health clinics. The models did not include pre-diagnostic loss to follow-up, patient costs, or patient perspectives on stool testing. Expanding diagnostic capacity to PHCs may improve access for more children and reduce follow-up visits, decreasing diagnostic delays and costs.

Strengths of this analysis include utilizing primary data on stool testing from the diagnostic accuracy study and parameters from the TB Speed decentralization study. This is one of the first studies to assess the use and potential cost-effectiveness of the new TDAs, and the models accounted for complexity in the clinical pathway, including high-risk children and availability of stool samples. Furthermore, we consulted expert opinion and conducted multiple sensitivity analyses of clinical parameters to investigate the impact of uncertainty on the model outputs.

## Conclusions

In summary, our findings show that the Simple One-Step was the least costly stool processing method. While stool testing was only cost-effective under some conditions, implementation at primary health centers has lower costs in relation to lives saved than evaluation only at district hospitals. Key drivers of cost effectiveness are TB prevalence and other factors including patient characteristics and initial screening. Lower cost molecular diagnostics that have similar or higher sensitivity should be explored for stool-based TB testing at the point-of-care to improve access and cost-effectiveness for young children.

## Supplementary Information

Below is the link to the electronic supplementary material.

Supplementary Material 1: Additional file 1_Decision tree model.pdf This file contains the decision-tree models used for the cost-effectiveness analysis.

Supplementary Material 2: Additional file 2_CHEERS Checklist.pdf This file contains a checklist for the Consolidated Health Economic Evaluation Reporting Standards guidance.

Supplementary Material 3: Additional file 3_Time and motion results.xlsx This file contains the results of the time and motion study.

## Data Availability

All data generated and analyzed during this study are included in this published article and its supplementary information files.

## Abbreviations

CXR: Chest X-ray
DH: District hospital
ICER: Incremental cost-effectiveness ratio
I$: International dollars
LAM: Lipoarabinomannan
LYS: Llife years saved
OSF: Optimized Sucrose Flotation
PHC: Primary health clinic
SPK: Stool Processing Kit
SOS: Simple One-Step
TB: Tuberculosis
TDA: Treatment decision algorithm
WHO: World Health Organization
WTP: Willingness to pay
