# Supplementary material for: A cost-effectiveness analysis of novel stool processing methods for diagnosis of tuberculosis in children under 5 years of age in Uganda
Source: BMC Health Serv Res. 2025 Oct 13;25:1353. doi: 10.1186/s12913-025-13546-3 (PMC12519833; doi:10.1186/s12913-025-13546-3)
Supplement: Supplementary file 3 — Supplementary Material 3: Additional file 3_Time and motion results.xlsx This file contains the results of the time and motion study. [file 12913_2025_13546_MOESM3_ESM.pdf]

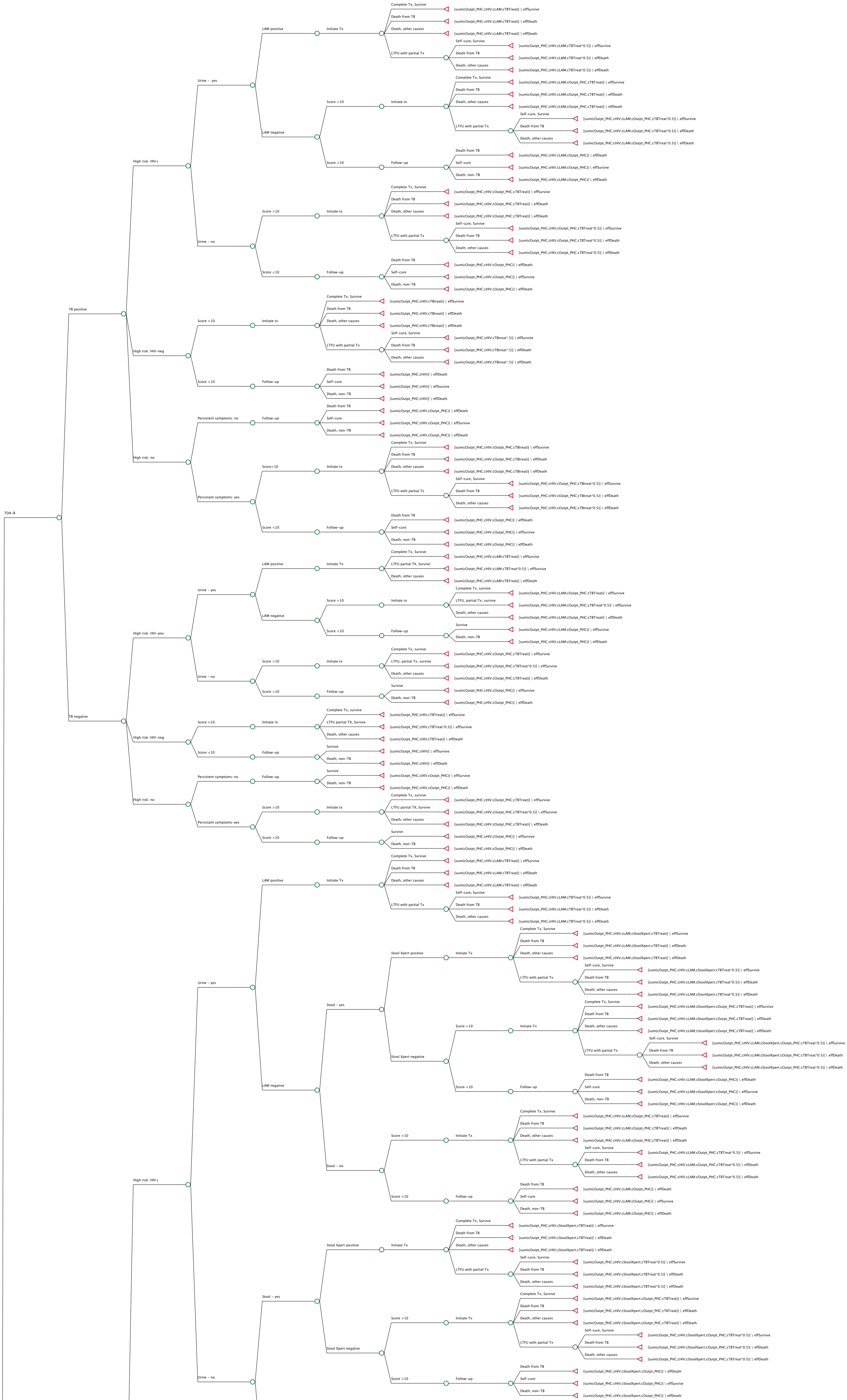
TDA-8
TB positive
High risk: HIV+
Urine - yes
LAM positive
Initiate Tx
Complete Tx, Survive
[sum(cOutpt_PHC;cHIV;cLAM;cTBTreat)] \ effSurvive
Death from TB
[sum(cOutpt_PHC;cHIV;cLAM;cTBTreat)] \ effDeath
Death, other causes
[sum(cOutpt_PHC;cHIV;cLAM;cTBTreat)] \ effDeath
LTFU with partial Tx
Self-cure, Survive
[sum(cOutpt_PHC;cHIV;cLAM;cTBTreat*0.5)] \ effSurvive
Death from TB
[sum(cOutpt_PHC;cHIV;cLAM;cTBTreat*0.5)] \ effDeath
Death, other causes
[sum(cOutpt_PHC;cHIV;cLAM;cTBTreat*0.5)] \ effDeath
LAM negative
Score >10
Initiate tx
Complete Tx, Survive
[sum(cOutpt_PHC;cHIV;cLAM;cOutpt_PHC;cTBTreat)] \ effSurvive
Death from TB
[sum(cOutpt_PHC;cHIV;cLAM;cOutpt_PHC;cTBTreat)] \ effDeath
Death, other causes
[sum(cOutpt_PHC;cHIV;cLAM;cOutpt_PHC;cTBTreat)] \ effDeath
LTFU with partial Tx
Self-cure, Survive
[sum(cOutpt_PHC;cHIV;cLAM;cOutpt_PHC;cTBTreat*0.5)] \ effSurvive
Death from TB
[sum(cOutpt_PHC;cHIV;cLAM;cOutpt_PHC;cTBTreat*0.5)] \ effDeath
Death, other causes
[sum(cOutpt_PHC;cHIV;cLAM;cOutpt_PHC;cTBTreat*0.5)] \ effDeath
Score <10
Follow-up
Death from TB
[sum(cOutpt_PHC;cHIV;cLAM;cOutpt_PHC)] \ effDeath
Self-cure
[sum(cOutpt_PHC;cHIV;cLAM;cOutpt_PHC)] \ effSurvive
Death, non-TB
[sum(cOutpt_PHC;cHIV;cLAM;cOutpt_PHC)] \ effDeath
Urine - no
Score >10
Initiate tx
Complete Tx, Survive
[sum(cOutpt_PHC;cHIV;cOutpt_PHC;cTBTreat)] \ effSurvive
Death from TB
[sum(cOutpt_PHC;cHIV;cOutpt_PHC;cTBTreat)] \ effDeath
Death, other causes
[sum(cOutpt_PHC;cHIV;cOutpt_PHC;cTBTreat)] \ effDeath
LTFU with partial Tx
Self-cure, Survive
[sum(cOutpt_PHC;cHIV;cOutpt_PHC;cTBTreat*0.5)] \ effSurvive
Death from TB
[sum(cOutpt_PHC;cHIV;cOutpt_PHC;cTBTreat*0.5)] \ effDeath
Death, other causes
[sum(cOutpt_PHC;cHIV;cOutpt_PHC;cTBTreat*0.5)] \ effDeath
Score <10
Follow-up
Death from TB
[sum(cOutpt_PHC;cHIV;cOutpt_PHC)] \ effDeath
Self-cure
[sum(cOutpt_PHC;cHIV;cOutpt_PHC)] \ effSurvive
Death, non-TB
[sum(cOutpt_PHC;cHIV;cOutpt_PHC)] \ effDeath
High risk: HIV-neg
Score >10
Initiate tx
Complete Tx, Survive
[sum(cOutpt_PHC;cHIV;cTBtreat)] \ effSurvive
Death from TB
[sum(cOutpt_PHC;cHIV;cTBtreat)] \ effDeath
Death, other causes
[sum(cOutpt_PHC;cHIV;cTBtreat)] \ effDeath
LTFU with partial Tx
Self-cure, Survive
[sum(cOutpt_PHC;cHIV;cTBtreat*.5)] \ effSurvive
Death from TB
[sum(cOutpt_PHC;cHIV;cTBtreat*.5)] \ effDeath
Death, other causes
[sum(cOutpt_PHC;cHIV;cTBtreat*.5)] \ effDeath
Score <10
Follow-up
Death from TB
[sum(cOutpt_PHC;cHIV)] \ effDeath
Self-cure
[sum(cOutpt_PHC;cHIV)] \ effSurvive
Death, non-TB
[sum(cOutpt_PHC;cHIV)] \ effDeath
High risk: no
Persistent symptoms: no
Follow-up
Death from TB
[sum(cOutpt_PHC;cHIV;cOutpt_PHC)] \ effDeath
Self-cure
[sum(cOutpt_PHC;cHIV;cOutpt_PHC)] \ effSurvive
Death, non-TB
[sum(cOutpt_PHC;cHIV;cOutpt_PHC)] \ effDeath
Persistant symptoms: yes
Score>10
Initiate tx
Complete Tx, Survive
[sum(cOutpt_PHC;cHIV;cOutpt_PHC;cTBtreat)] \ effSurvive
Death from TB
[sum(cOutpt_PHC;cHIV;cOutpt_PHC;cTBtreat)] \ effDeath
Death, other causes
[sum(cOutpt_PHC;cHIV;cOutpt_PHC;cTBtreat)] \ effDeath
LTFU with partial Tx
Self-cure, Survive
[sum(cOutpt_PHC;cHIV;cOutpt_PHC;cTBtreat*0.5)] \ effSurvive
Death from TB
[sum(cOutpt_PHC;cHIV;cOutpt_PHC;cTBtreat*0.5)] \ effDeath
Death, other causes
[sum(cOutpt_PHC;cHIV;cOutpt_PHC;cTBtreat*0.5)] \ effDeath
Score <10
Follow-up
Death from TB
[sum(cOutpt_PHC;cHIV;cOutpt_PHC)] \ effDeath
Self-cure
[sum(cOutpt_PHC;cHIV;cOutpt_PHC)] \ effSurvive
Death, non-TB
[sum(cOutpt_PHC;cHIV;cOutpt_PHC)] \ effDeath
TB negative
High risk: HIV-pos
Urine - yes
LAM positive
Initiate Tx
Complete Tx, Survive
[sum(cOutpt_PHC;cHIV;cLAM;cTBTreat)] \ effSurvive
LTFU partial TX, Survive
[sum(cOutpt_PHC;cHIV;cLAM;cTBTreat*0.5)] \ effSurvive
Death, other causes
[sum(cOutpt_PHC;cHIV;cLAM;cTBTreat)] \ effDeath
LAM negative
Score >10
Initiate tx
Complete Tx, survive
[sum(cOutpt_PHC;cHIV;cLAM;cOutpt_PHC;cTBTreat)] \ effSurvive
LTFU, partial Tx, survive
[sum(cOutpt_PHC;cHIV;cLAM;cOutpt_PHC;cTBTreat*0.5)] \ effSurvive
Death, other causes
[sum(cOutpt_PHC;cHIV;cLAM;cOutpt_PHC;cTBTreat)] \ effDeath
Score <10
Follow-up
Survive
[sum(cOutpt_PHC;cHIV;cLAM;cOutpt_PHC)] \ effSurvive
Death, non-TB
[sum(cOutpt_PHC;cHIV;cLAM;cOutpt_PHC)] \ effDeath
Urine - no
Score >10
Initiate tx
Complete Tx, survive
[sum(cOutpt_PHC;cHIV;cOutpt_PHC;cTBTreat)] \ effSurvive
LTFU, partial Tx, survive
[sum(cOutpt_PHC;cHIV;cOutpt_PHC;cTBTreat*0.5)] \ effSurvive
Death, other causes
[sum(cOutpt_PHC;cHIV;cOutpt_PHC;cTBTreat)] \ effDeath
Score <10
Follow-up
Survive
[sum(cOutpt_PHC;cHIV;cOutpt_PHC)] \ effSurvive
Death, non-TB
[sum(cOutpt_PHC;cHIV;cOutpt_PHC)] \ effDeath
High risk: HIV-neg
Score >10
Initiate tx
Complete Tx, survive
[sum(cOutpt_PHC;cHIV;cTBTreat)] \ effSurvive
LTFU partial TX, Survive
[sum(cOutpt_PHC;cHIV;cTBTreat*0.5)] \ effSurvive
Death, other causes
[sum(cOutpt_PHC;cHIV;cTBTreat)] \ effDeath
Score <10
Follow-up
Survive
[sum(cOutpt_PHC;cHIV)] \ effSurvive
Death, non-TB
[sum(cOutpt_PHC;cHIV)] \ effDeath
High risk: no
Persistent symptoms-no
Follow-up
Survive
[sum(cOutpt_PHC;cHIV;cOutpt_PHC)] \ effSurvive
Death, non-TB
[sum(cOutpt_PHC;cHIV;cOutpt_PHC)] \ effDeath
Persistant symptoms-yes
Score >10
Initiate tx
Complete Tx, survive
[sum(cOutpt_PHC;cHIV;cOutpt_PHC;cTBTreat)] \ effSurvive
LTFU partial TX, Survive
[sum(cOutpt_PHC;cHIV;cOutpt_PHC;cTBTreat*0.5)] \ effSurvive
Death, other causes
[sum(cOutpt_PHC;cHIV;cOutpt_PHC;cTBTreat)] \ effDeath
Score <10
Follow-up
Survive
[sum(cOutpt_PHC;cHIV;cOutpt_PHC)] \ effSurvive
Death, non-TB
[sum(cOutpt_PHC;cHIV;cOutpt_PHC)] \ effDeath
High risk: HIV+
Urine - yes
LAM positive
Initiate Tx
Complete Tx, Survive
[sum(cOutpt_PHC;cHIV;cLAM;cTBTreat)] \ effSurvive
Death from TB
[sum(cOutpt_PHC;cHIV;cLAM;cTBTreat)] \ effDeath
Death, other causes
[sum(cOutpt_PHC;cHIV;cLAM;cTBTreat)] \ effDeath
LTFU with partial Tx
Self-cure, Survive
[sum(cOutpt_PHC;cHIV;cLAM;cTBTreat*0.5)] \ effSurvive
Death from TB
[sum(cOutpt_PHC;cHIV;cLAM;cTBTreat*0.5)] \ effDeath
Death, other causes
[sum(cOutpt_PHC;cHIV;cLAM;cTBTreat*0.5)] \ effDeath
LAM negative
Stool - yes
Stool Xpert positive
Initiate Tx
Complete Tx, Survive
[sum(cOutpt_PHC;cHIV;cLAM;cStoolXpert;cTBTreat)] \ effSurvive
Death from TB
[sum(cOutpt_PHC;cHIV;cLAM;cStoolXpert;cTBTreat)] \ effDeath
Death, other causes
[sum(cOutpt_PHC;cHIV;cLAM;cStoolXpert;cTBTreat)] \ effDeath
LTFU with partial Tx
Self-cure, Survive
[sum(cOutpt_PHC;cHIV;cLAM;cStoolXpert;cTBTreat*0.5)] \ effSurvive
Death from TB
[sum(cOutpt_PHC;cHIV;cLAM;cStoolXpert;cTBTreat*0.5)] \ effDeath
Death, other causes
[sum(cOutpt_PHC;cHIV;cLAM;cStoolXpert;cTBTreat*0.5)] \ effDeath
Stool Xpert negative
Score >10
Initiate Tx
Complete Tx, Survive
[sum(cOutpt_PHC;cHIV;cLAM;cStoolXpert;cOutpt_PHC;cTBTreat)] \ effSurvive
Death from TB
[sum(cOutpt_PHC;cHIV;cLAM;cStoolXpert;cOutpt_PHC;cTBTreat)] \ effDeath
Death, other causes
[sum(cOutpt_PHC;cHIV;cLAM;cStoolXpert;cOutpt_PHC;cTBTreat)] \ effDeath
LTFU with partial Tx
Self-cure, Survive
[sum(cOutpt_PHC;cHIV;cLAM;cStoolXpert;cOutpt_PHC;cTBTreat*0.5)] \ effSurvive
Death from TB
[sum(cOutpt_PHC;cHIV;cLAM;cStoolXpert;cOutpt_PHC;cTBTreat*0.5)] \ effDeath
Death, other causes
[sum(cOutpt_PHC;cHIV;cLAM;cStoolXpert;cOutpt_PHC;cTBTreat*0.5)] \ effDeath
Score <10
Follow-up
Death from TB
[sum(cOutpt_PHC;cHIV;cLAM;cStoolXpert;cOutpt_PHC)] \ effDeath
Self-cure
[sum(cOutpt_PHC;cHIV;cLAM;cStoolXpert;cOutpt_PHC)] \ effSurvive
Death, non-TB
[sum(cOutpt_PHC;cHIV;cLAM;cStoolXpert;cOutpt_PHC)] \ effDeath
Stool - no
Score >10
Initiate Tx
Complete Tx, Survive
[sum(cOutpt_PHC;cHIV;cLAM;cOutpt_PHC;cTBTreat)] \ effSurvive
Death from TB
[sum(cOutpt_PHC;cHIV;cLAM;cOutpt_PHC;cTBTreat)] \ effDeath
Death, other causes
[sum(cOutpt_PHC;cHIV;cLAM;cOutpt_PHC;cTBTreat)] \ effDeath
LTFU with partial Tx
Self-cure, Survive
[sum(cOutpt_PHC;cHIV;cLAM;cOutpt_PHC;cTBTreat*0.5)] \ effSurvive
Death from TB
[sum(cOutpt_PHC;cHIV;cLAM;cOutpt_PHC;cTBTreat*0.5)] \ effDeath
Death, other causes
[sum(cOutpt_PHC;cHIV;cLAM;cOutpt_PHC;cTBTreat*0.5)] \ effDeath
Score <10
Follow-up
Death from TB
[sum(cOutpt_PHC;cHIV;cLAM;cOutpt_PHC)] \ effDeath
Self-cure
[sum(cOutpt_PHC;cHIV;cLAM;cOutpt_PHC)] \ effSurvive
Death, non-TB
[sum(cOutpt_PHC;cHIV;cLAM;cOutpt_PHC)] \ effDeath
Urine - no
Stool - yes
Stool Xpert positive
Initiate Tx
Complete Tx, Survive
[sum(cOutpt_PHC;cHIV;cStoolXpert;cTBTreat)] \ effSurvive
Death from TB
[sum(cOutpt_PHC;cHIV;cStoolXpert;cTBTreat)] \ effDeath
Death, other causes
[sum(cOutpt_PHC;cHIV;cStoolXpert;cTBTreat)] \ effDeath
LTFU with partial Tx
Self-cure, Survive
[sum(cOutpt_PHC;cHIV;cStoolXpert;cTBTreat*0.5)] \ effSurvive
Death from TB
[sum(cOutpt_PHC;cHIV;cStoolXpert;cTBTreat*0.5)] \ effDeath
Death, other causes
[sum(cOutpt_PHC;cHIV;cStoolXpert;cTBTreat*0.5)] \ effDeath
Stool Xpert negative
Score >10
Initiate Tx
Complete Tx, Survive
[sum(cOutpt_PHC;cHIV;cStoolXpert;cOutpt_PHC;cTBTreat)] \ effSurvive
Death from TB
[sum(cOutpt_PHC;cHIV;cStoolXpert;cOutpt_PHC;cTBTreat)] \ effDeath
Death, other causes
[sum(cOutpt_PHC;cHIV;cStoolXpert;cOutpt_PHC;cTBTreat)] \ effDeath
LTFU with partial Tx
Self-cure, Survive
[sum(cOutpt_PHC;cHIV;cStoolXpert;cOutpt_PHC;cTBTreat*0.5)] \ effSurvive
Death from TB
[sum(cOutpt_PHC;cHIV;cStoolXpert;cOutpt_PHC;cTBTreat*0.5)] \ effDeath
Death, other causes
[sum(cOutpt_PHC;cHIV;cStoolXpert;cOutpt_PHC;cTBTreat*0.5)] \ effDeath
Score <10
Follow-up
Death from TB
[sum(cOutpt_PHC;cHIV;cStoolXpert;cOutpt_PHC)] \ effDeath
Self-cure
[sum(cOutpt_PHC;cHIV;cStoolXpert;cOutpt_PHC)] \ effSurvive
Death, non-TB
[sum(cOutpt_PHC;cHIV;cStoolXpert;cOutpt_PHC)] \ effDeath

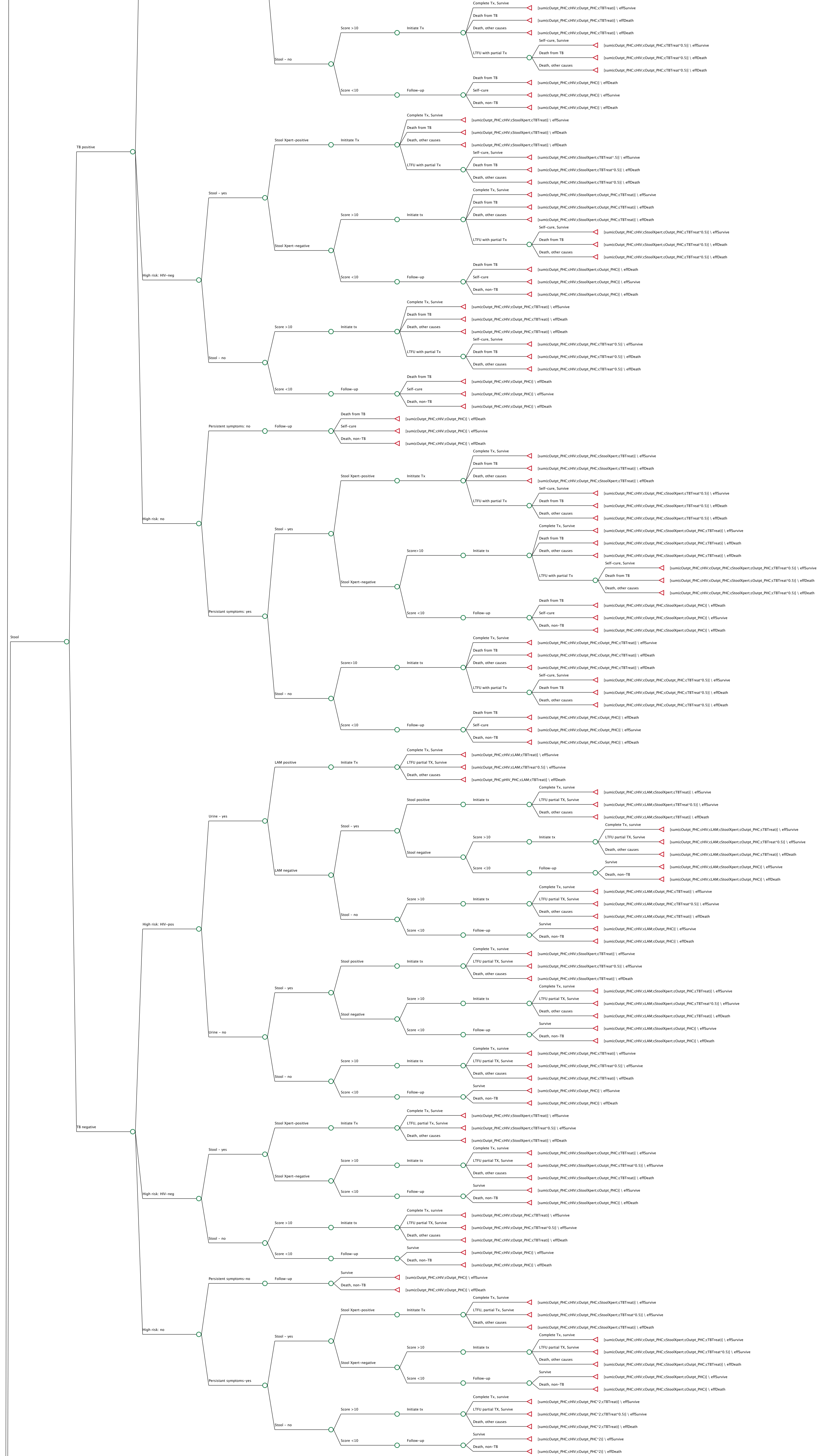

Complete Tx, Survive
[sum(cOutpt_PHC;cHIV;cOutpt_PHC;cTBTreat)] \ effSurvive
Death from TB
[sum(cOutpt_PHC;cHIV;cOutpt_PHC;cTBTreat)] \ effDeath
Score >10
Initiate Tx
Death, other causes
[sum(cOutpt_PHC;cHIV;cOutpt_PHC;cTBTreat)] \ effDeath
Self-cure, Survive
[sum(cOutpt_PHC;cHIV;cOutpt_PHC;cTBTreat*0.5)] \ effSurvive
LTFU with partial Tx
Death from TB
[sum(cOutpt_PHC;cHIV;cOutpt_PHC;cTBTreat*0.5)] \ effDeath
Stool - no
Death, other causes
[sum(cOutpt_PHC;cHIV;cOutpt_PHC;cTBTreat*0.5)] \ effDeath
Death from TB
[sum(cOutpt_PHC;cHIV;cOutpt_PHC)] \ effDeath
Score <10
Follow-up
Self-cure
[sum(cOutpt_PHC;cHIV;cOutpt_PHC)] \ effSurvive
Death, non-TB
[sum(cOutpt_PHC;cHIV;cOutpt_PHC)] \ effDeath
Complete Tx, Survive
[sum(cOutpt_PHC;cHIV;cStoolXpert;cTBTreat)] \ effSurvive
Death from TB
[sum(cOutpt_PHC;cHIV;cStoolXpert;cTBTreat)] \ effDeath
TB positive
Stool Xpert-positive
Inititate Tx
Death, other causes
[sum(cOutpt_PHC;cHIV;cStoolXpert;cTBTreat)] \ effDeath
Self-cure, Survive
[sum(cOutpt_PHC;cHIV;cStoolXpert;cTBTreat*.5)] \ effSurvive
LTFU with partial Tx
Death from TB
[sum(cOutpt_PHC;cHIV;cStoolXpert;cTBTreat*0.5)] \ effDeath
Death, other causes
[sum(cOutpt_PHC;cHIV;cStoolXpert;cTBTreat*0.5)] \ effDeath
Complete Tx, Survive
Stool - yes
[sum(cOutpt_PHC;cHIV;cStoolXpert;cOutpt_PHC;cTBTreat)] \ effSurvive
Death from TB
[sum(cOutpt_PHC;cHIV;cStoolXpert;cOutpt_PHC;cTBTreat)] \ effDeath
Score >10
Initiate tx
Death, other causes
[sum(cOutpt_PHC;cHIV;cStoolXpert;cOutpt_PHC;cTBTreat)] \ effDeath
Self-cure, Survive
[sum(cOutpt_PHC;cHIV;cStoolXpert;cOutpt_PHC;cTBTreat*0.5)] \ effSurvive
LTFU with partial Tx
Death from TB
[sum(cOutpt_PHC;cHIV;cStoolXpert;cOutpt_PHC;cTBTreat*0.5)] \ effDeath
Stool Xpert-negative
Death, other causes
[sum(cOutpt_PHC;cHIV;cStoolXpert;cOutpt_PHC;cTBTreat*0.5)] \ effDeath
Death from TB
[sum(cOutpt_PHC;cHIV;cStoolXpert;cOutpt_PHC)] \ effDeath
Score <10
Follow-up
Self-cure
High risk: HIV-neg
[sum(cOutpt_PHC;cHIV;cStoolXpert;cOutpt_PHC)] \ effSurvive
Death, non-TB
[sum(cOutpt_PHC;cHIV;cStoolXpert;cOutpt_PHC)] \ effDeath
Complete Tx, Survive
[sum(cOutpt_PHC;cHIV;cOutpt_PHC;cTBTreat)] \ effSurvive
Death from TB
[sum(cOutpt_PHC;cHIV;cOutpt_PHC;cTBTreat)] \ effDeath
Score >10
Initiate tx
Death, other causes
[sum(cOutpt_PHC;cHIV;cOutpt_PHC;cTBTreat)] \ effDeath
Self-cure, Survive
[sum(cOutpt_PHC;cHIV;cOutpt_PHC;cTBTreat*0.5)] \ effSurvive
LTFU with partial Tx
Death from TB
[sum(cOutpt_PHC;cHIV;cOutpt_PHC;cTBTreat*0.5)] \ effDeath
Stool - no
Death, other causes
[sum(cOutpt_PHC;cHIV;cOutpt_PHC;cTBTreat*0.5)] \ effDeath
Death from TB
[sum(cOutpt_PHC;cHIV;cOutpt_PHC)] \ effDeath
Score <10
Follow-up
Self-cure
[sum(cOutpt_PHC;cHIV;cOutpt_PHC)] \ effSurvive
Death, non-TB
[sum(cOutpt_PHC;cHIV;cOutpt_PHC)] \ effDeath
Death from TB
[sum(cOutpt_PHC;cHIV;cOutpt_PHC)] \ effDeath
Persistent symptoms: no
Follow-up
Self-cure
[sum(cOutpt_PHC;cHIV;cOutpt_PHC)] \ effSurvive
Death, non-TB
[sum(cOutpt_PHC;cHIV;cOutpt_PHC)] \ effDeath
Complete Tx, Survive
[sum(cOutpt_PHC;cHIV;cOutpt_PHC;cStoolXpert;cTBTreat)] \ effSurvive
Death from TB
[sum(cOutpt_PHC;cHIV;cOutpt_PHC;cStoolXpert;cTBTreat)] \ effDeath
Stool Xpert-positive
Initiate Tx
Death, other causes
[sum(cOutpt_PHC;cHIV;cOutpt_PHC;cStoolXpert;cTBTreat)] \ effDeath
Self-cure, Survive
[sum(cOutpt_PHC;cHIV;cOutpt_PHC;cStoolXpert;cTBTreat*0.5)] \ effSurvive
LTFU with partial Tx
Death from TB
[sum(cOutpt_PHC;cHIV;cOutpt_PHC;cStoolXpert;cTBTreat*0.5)] \ effDeath
High risk: no
Death, other causes
[sum(cOutpt_PHC;cHIV;cOutpt_PHC;cStoolXpert;cTBTreat*0.5)] \ effDeath
Stool - yes
Complete Tx, Survive
[sum(cOutpt_PHC;cHIV;cOutpt_PHC;cStoolXpert;cOutpt_PHC;cTBTreat)] \ effSurvive
Death from TB
[sum(cOutpt_PHC;cHIV;cOutpt_PHC;cStoolXpert;cOutpt_PHC;cTBTreat)] \ effDeath
Score>10
Initiate tx
Death, other causes
[sum(cOutpt_PHC;cHIV;cOutpt_PHC;cStoolXpert;cOutpt_PHC;cTBTreat)] \ effDeath
Self-cure, Survive
[sum(cOutpt_PHC;cHIV;cOutpt_PHC;cStoolXpert;cOutpt_PHC;cTBTreat*0.5)] \ effSurvive
LTFU with partial Tx
Death from TB
[sum(cOutpt_PHC;cHIV;cOutpt_PHC;cStoolXpert;cOutpt_PHC;cTBTreat*0.5)] \ effDeath
Stool Xpert-negative
Death, other causes
[sum(cOutpt_PHC;cHIV;cOutpt_PHC;cStoolXpert;cOutpt_PHC;cTBTreat*0.5)] \ effDeath
Death from TB
[sum(cOutpt_PHC;cHIV;cOutpt_PHC;cStoolXpert;cOutpt_PHC)] \ effDeath
Score <10
Follow-up
Self-cure
Persistant symptoms: yes
[sum(cOutpt_PHC;cHIV;cOutpt_PHC;cStoolXpert;cOutpt_PHC)] \ effSurvive
Death, non-TB
[sum(cOutpt_PHC;cHIV;cOutpt_PHC;cStoolXpert;cOutpt_PHC)] \ effDeath
Stool
Complete Tx, Survive
[sum(cOutpt_PHC;cHIV;cOutpt_PHC;cOutpt_PHC;cTBTreat)] \ effSurvive
Death from TB
[sum(cOutpt_PHC;cHIV;cOutpt_PHC;cOutpt_PHC;cTBTreat)] \ effDeath
Score>10
Initiate tx
Death, other causes
[sum(cOutpt_PHC;cHIV;cOutpt_PHC;cOutpt_PHC;cTBTreat)] \ effDeath
Self-cure, Survive
[sum(cOutpt_PHC;cHIV;cOutpt_PHC;cOutpt_PHC;cTBTreat*0.5)] \ effSurvive
LTFU with partial Tx
Death from TB
[sum(cOutpt_PHC;cHIV;cOutpt_PHC;cOutpt_PHC;cTBTreat*0.5)] \ effDeath
Stool - no
Death, other causes
[sum(cOutpt_PHC;cHIV;cOutpt_PHC;cOutpt_PHC;cTBTreat*0.5)] \ effDeath
Death from TB
[sum(cOutpt_PHC;cHIV;cOutpt_PHC;cOutpt_PHC)] \ effDeath
Score <10
Follow-up
Self-cure
[sum(cOutpt_PHC;cHIV;cOutpt_PHC;cOutpt_PHC)] \ effSurvive
Death, non-TB
[sum(cOutpt_PHC;cHIV;cOutpt_PHC;cOutpt_PHC)] \ effDeath
Complete Tx, Survive
[sum(cOutpt_PHC;cHIV;cLAM;cTBTreat)] \ effSurvive
LAM positive
Initiate Tx
LTFU partial TX, Survive
[sum(cOutpt_PHC;cHIV;cLAM;cTBTreat*0.5)] \ effSurvive
Death, other causes
[sum(cOutpt_PHC;pHIV_PHC;cLAM;cTBTreat)] \ effDeath
Complete Tx, survive
[sum(cOutpt_PHC;cHIV;cLAM;cStoolXpert;cTBTreat)] \ effSurvive
Stool positive
Initiate tx
LTFU partial TX, Survive
[sum(cOutpt_PHC;cHIV;cLAM;cStoolXpert;cTBTreat*0.5)] \ effSurvive
Death, other causes
[sum(cOutpt_PHC;cHIV;cLAM;cStoolXpert;cTBTreat)] \ effDeath
Urine - yes
Complete Tx, survive
Stool - yes
[sum(cOutpt_PHC;cHIV;cLAM;cStoolXpert;cOutpt_PHC;cTBTreat)] \ effSurvive
Score >10
Initiate tx
LTFU partial TX, Survive
[sum(cOutpt_PHC;cHIV;cLAM;cStoolXpert;cOutpt_PHC;cTBTreat*0.5)] \ effSurvive
Death, other causes
[sum(cOutpt_PHC;cHIV;cLAM;cStoolXpert;cOutpt_PHC;cTBTreat)] \ effDeath
Stool negative
Survive
[sum(cOutpt_PHC;cHIV;cLAM;cStoolXpert;cOutpt_PHC)] \ effSurvive
Score <10
Follow-up
Death, non-TB
[sum(cOutpt_PHC;cHIV;cLAM;cStoolXpert;cOutpt_PHC)] \ effDeath
LAM negative
Complete Tx, survive
[sum(cOutpt_PHC;cHIV;cLAM;cOutpt_PHC;cTBTreat)] \ effSurvive
Score >10
Initiate tx
LTFU partial TX, Survive
[sum(cOutpt_PHC;cHIV;cLAM;cOutpt_PHC;cTBTreat*0.5)] \ effSurvive
Stool - no
Death, other causes
[sum(cOutpt_PHC;cHIV;cLAM;cOutpt_PHC;cTBTreat)] \ effDeath
Survive
[sum(cOutpt_PHC;cHIV;cLAM;cOutpt_PHC)] \ effSurvive
High risk: HIV-pos
Score <10
Follow-up
Death, non-TB
[sum(cOutpt_PHC;cHIV;cLAM;cOutpt_PHC)] \ effDeath
Complete Tx, survive
[sum(cOutpt_PHC;cHIV;cStoolXpert;cTBTreat)] \ effSurvive
Stool positive
Initiate tx
LTFU partial TX, Survive
[sum(cOutpt_PHC;cHIV;cStoolXpert;cTBTreat*0.5)] \ effSurvive
Death, other causes
[sum(cOutpt_PHC;cHIV;cStoolXpert;cTBTreat)] \ effDeath
Stool - yes
Complete Tx, survive
[sum(cOutpt_PHC;cHIV;cLAM;cStoolXpert;cOutpt_PHC;cTBTreat)] \ effSurvive
Score >10
Initiate tx
LTFU partial TX, Survive
[sum(cOutpt_PHC;cHIV;cLAM;cStoolXpert;cOutpt_PHC;cTBTreat*0.5)] \ effSurvive
Stool negative
Death, other causes
[sum(cOutpt_PHC;cHIV;cLAM;cStoolXpert;cOutpt_PHC;cTBTreat)] \ effDeath
Survive
[sum(cOutpt_PHC;cHIV;cLAM;cStoolXpert;cOutpt_PHC)] \ effSurvive
Score <10
Follow-up
Urine - no
Death, non-TB
[sum(cOutpt_PHC;cHIV;cLAM;cStoolXpert;cOutpt_PHC)] \ effDeath
Complete Tx, survive
[sum(cOutpt_PHC;cHIV;cOutpt_PHC;cTBTreat)] \ effSurvive
Score >10
Initiate tx
LTFU partial TX, Survive
[sum(cOutpt_PHC;cHIV;cOutpt_PHC;cTBTreat*0.5)] \ effSurvive
Death, other causes
Stool - no
[sum(cOutpt_PHC;cHIV;cOutpt_PHC;cTBTreat)] \ effDeath
Survive
[sum(cOutpt_PHC;cHIV;cOutpt_PHC)] \ effSurvive
Score <10
Follow-up
Death, non-TB
[sum(cOutpt_PHC;cHIV;cOutpt_PHC)] \ effDeath
Complete Tx, Survive
[sum(cOutpt_PHC;cHIV;cStoolXpert;cTBTreat)] \ effSurvive
Stool Xpert-positive
Initiate Tx
LTFU, partial Tx, Survive
[sum(cOutpt_PHC;cHIV;cStoolXpert;cTBTreat*0.5)] \ effSurvive
TB negative
Death, other causes
[sum(cOutpt_PHC;cHIV;cStoolXpert;cTBTreat)] \ effDeath
Complete Tx, survive
Stool - yes
[sum(cOutpt_PHC;cHIV;cStoolXpert;cOutpt_PHC;cTBTreat)] \ effSurvive
Score >10
Initiate tx
LTFU partial TX, Survive
[sum(cOutpt_PHC;cHIV;cStoolXpert;cOutpt_PHC;cTBTreat*0.5)] \ effSurvive
Stool Xpert-negative
Death, other causes
[sum(cOutpt_PHC;cHIV;cStoolXpert;cOutpt_PHC;cTBTreat)] \ effDeath
Survive
[sum(cOutpt_PHC;cHIV;cStoolXpert;cOutpt_PHC)] \ effSurvive
Score <10
Follow-up
High risk: HIV-neg
Death, non-TB
[sum(cOutpt_PHC;cHIV;cStoolXpert;cOutpt_PHC)] \ effDeath
Complete Tx, survive
[sum(cOutpt_PHC;cHIV;cOutpt_PHC;cTBTreat)] \ effSurvive
Score >10
Initiate tx
LTFU partial TX, Survive
[sum(cOutpt_PHC;cHIV;cOutpt_PHC;cTBTreat*0.5)] \ effSurvive
Stool - no
Death, other causes
[sum(cOutpt_PHC;cHIV;cOutpt_PHC;cTBTreat)] \ effDeath
Survive
[sum(cOutpt_PHC;cHIV;cOutpt_PHC)] \ effSurvive
Score <10
Follow-up
Death, non-TB
[sum(cOutpt_PHC;cHIV;cOutpt_PHC)] \ effDeath
Survive
Persistent symptoms-no
Follow-up
[sum(cOutpt_PHC;cHIV;cOutpt_PHC)] \ effSurvive
Death, non-TB
[sum(cOutpt_PHC;cHIV;cOutpt_PHC)] \ effDeath
Complete Tx, Survive
[sum(cOutpt_PHC;cHIV;cOutpt_PHC;cStoolXpert;cTBTreat)] \ effSurvive
Stool Xpert-positive
Initiate Tx
LTFU, partial Tx, Survive
[sum(cOutpt_PHC;cHIV;cOutpt_PHC;cStoolXpert;cTBTreat*0.5)] \ effSurvive
Death, other causes
High risk: no
[sum(cOutpt_PHC;cHIV;cOutpt_PHC;cStoolXpert;cTBTreat)] \ effDeath
Complete Tx, survive
Stool - yes
[sum(cOutpt_PHC;cHIV;cOutpt_PHC;cStoolXpert;cOutpt_PHC;cTBTreat)] \ effSurvive
Score >10
Initiate tx
LTFU partial TX, Survive
[sum(cOutpt_PHC;cHIV;cOutpt_PHC;cStoolXpert;cOutpt_PHC;cTBTreat*0.5)] \ effSurvive
Death, other causes
Stool Xpert-negative
[sum(cOutpt_PHC;cHIV;cOutpt_PHC;cStoolXpert;cOutpt_PHC;cTBTreat)] \ effDeath
Survive
[sum(cOutpt_PHC;cHIV;cOutpt_PHC;cStoolXpert;cOutpt_PHC)] \ effSurvive
Score <10
Follow-up
Death, non-TB
Persistant symptoms-yes
[sum(cOutpt_PHC;cHIV;cOutpt_PHC;cStoolXpert;cOutpt_PHC)] \ effDeath
Complete Tx, survive
[sum(cOutpt_PHC;cHIV;cOutpt_PHC*2;cTBTreat)] \ effSurvive
Score >10
Initiate tx
LTFU partial TX, Survive
[sum(cOutpt_PHC;cHIV;cOutpt_PHC*2;cTBTreat*0.5)] \ effSurvive
Death, other causes
Stool - no
[sum(cOutpt_PHC;cHIV;cOutpt_PHC*2;cTBTreat)] \ effDeath
Survive
[sum(cOutpt_PHC;cHIV;cOutpt_PHC*2)] \ effSurvive
Score <10
Follow-up
Death, non-TB
[sum(cOutpt_PHC;cHIV;cOutpt_PHC*2)] \ effDeath

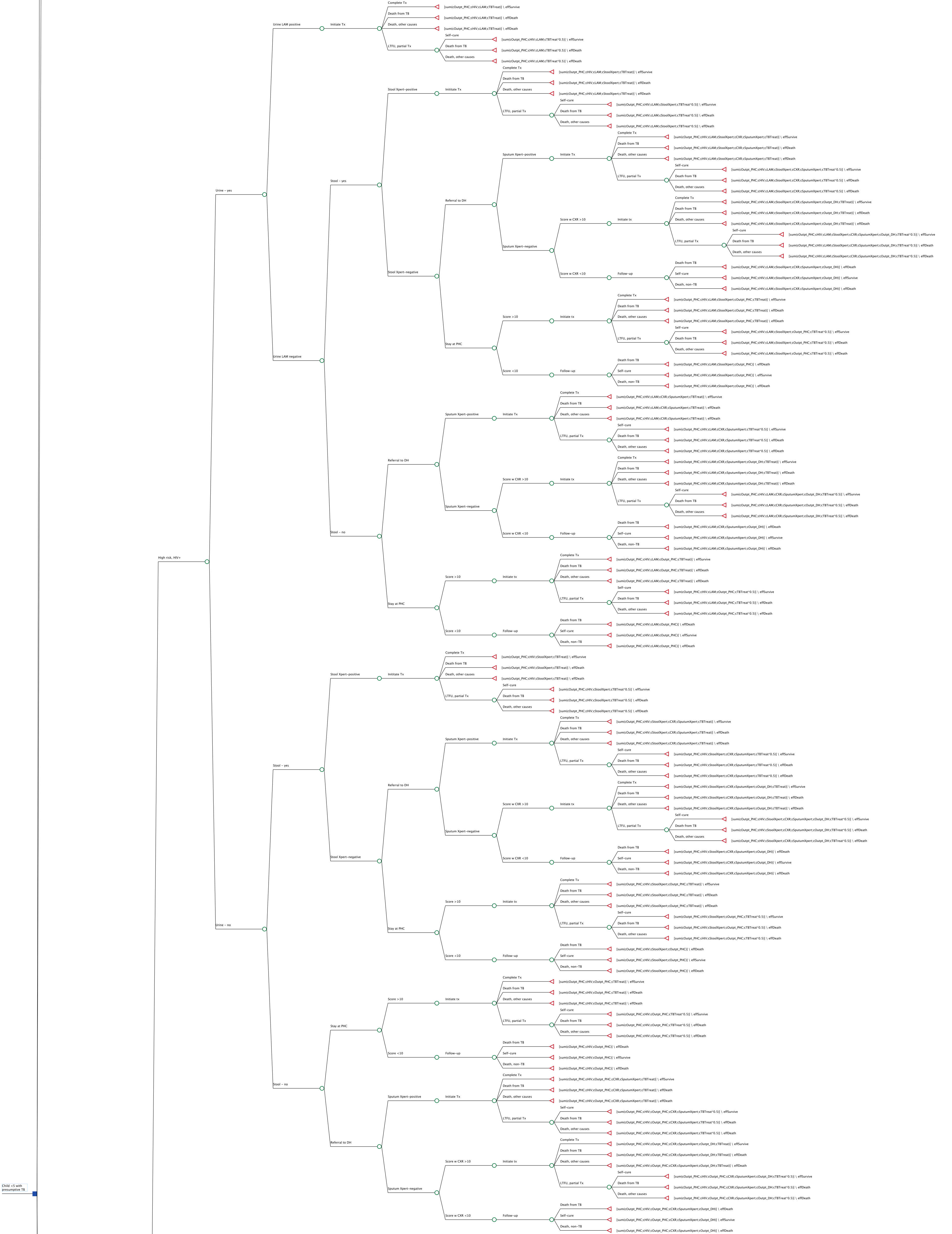

Complete Tx
[sum(cOutpt_PHC;cHIV;cLAM;cTBTreat)] \ effSurvive
Death from TB
[sum(cOutpt_PHC;cHIV;cLAM;cTBTreat)] \ effDeath
Urine LAM positive
Initiate Tx
Death, other causes
[sum(cOutpt_PHC;cHIV;cLAM;cTBTreat)] \ effDeath
Self-cure
[sum(cOutpt_PHC;cHIV;cLAM;cTBTreat*0.5)] \ effSurvive
LTFU, partial Tx
Death from TB
[sum(cOutpt_PHC;cHIV;cLAM;cTBTreat*0.5)] \ effDeath
Death, other causes
[sum(cOutpt_PHC;cHIV;cLAM;cTBTreat*0.5)] \ effDeath
Complete Tx
[sum(cOutpt_PHC;cHIV;cLAM;cStoolXpert;cTBTreat)] \ effSurvive
Death from TB
[sum(cOutpt_PHC;cHIV;cLAM;cStoolXpert;cTBTreat)] \ effDeath
Stool Xpert-positive
Inititate Tx
Death, other causes
[sum(cOutpt_PHC;cHIV;cLAM;cStoolXpert;cTBTreat)] \ effDeath
Self-cure
[sum(cOutpt_PHC;cHIV;cLAM;cStoolXpert;cTBTreat*0.5)] \ effSurvive
LTFU, partial Tx
Death from TB
[sum(cOutpt_PHC;cHIV;cLAM;cStoolXpert;cTBTreat*0.5)] \ effDeath
Death, other causes
[sum(cOutpt_PHC;cHIV;cLAM;cStoolXpert;cTBTreat*0.5)] \ effDeath
Complete Tx
[sum(cOutpt_PHC;cHIV;cLAM;cStoolXpert;cCXR;cSputumXpert;cTBTreat)] \ effSurvive
Death from TB
[sum(cOutpt_PHC;cHIV;cLAM;cStoolXpert;cCXR;cSputumXpert;cTBTreat)] \ effDeath
Sputum Xpert-positive
Initiate Tx
Death, other causes
[sum(cOutpt_PHC;cHIV;cLAM;cStoolXpert;cCXR;cSputumXpert;cTBTreat)] \ effDeath
Self-cure
[sum(cOutpt_PHC;cHIV;cLAM;cStoolXpert;cCXR;cSputumXpert;cTBTreat*0.5)] \ effSurvive
Death from TB
LTFU, partial Tx
[sum(cOutpt_PHC;cHIV;cLAM;cStoolXpert;cCXR;cSputumXpert;cTBTreat*0.5)] \ effDeath
Stool - yes
Death, other causes
[sum(cOutpt_PHC;cHIV;cLAM;cStoolXpert;cCXR;cSputumXpert;cTBTreat*0.5)] \ effDeath
Urine - yes
Complete Tx
Referral to DH
[sum(cOutpt_PHC;cHIV;cLAM;cStoolXpert;cCXR;cSputumXpert;cOutpt_DH;cTBTreat)] \ effSurvive
Death from TB
[sum(cOutpt_PHC;cHIV;cLAM;cStoolXpert;cCXR;cSputumXpert;cOutpt_DH;cTBTreat)] \ effDeath
Score w CXR >10
Initiate tx
Death, other causes
[sum(cOutpt_PHC;cHIV;cLAM;cStoolXpert;cCXR;cSputumXpert;cOutpt_DH;cTBTreat)] \ effDeath
Self-cure
[sum(cOutpt_PHC;cHIV;cLAM;cStoolXpert;cCXR;cSputumXpert;cOutpt_DH;cTBTreat*0.5)] \ effSurvive
LTFU, partial Tx
Death from TB
[sum(cOutpt_PHC;cHIV;cLAM;cStoolXpert;cCXR;cSputumXpert;cOutpt_DH;cTBTreat*0.5)] \ effDeath
Sputum Xpert-negative
Death, other causes
[sum(cOutpt_PHC;cHIV;cLAM;cStoolXpert;cCXR;cSputumXpert;cOutpt_DH;cTBTreat*0.5)] \ effDeath
Death from TB
[sum(cOutpt_PHC;cHIV;cLAM;cStoolXpert;cCXR;cSputumXpert;cOutpt_DH)] \ effDeath
Stool Xpert-negative
Score w CXR <10
Follow-up
Self-cure
[sum(cOutpt_PHC;cHIV;cLAM;cStoolXpert;cCXR;cSputumXpert;cOutpt_DH)] \ effSurvive
Death, non-TB
[sum(cOutpt_PHC;cHIV;cLAM;cStoolXpert;cCXR;cSputumXpert;cOutpt_DH)] \ effDeath
Complete Tx
[sum(cOutpt_PHC;cHIV;cLAM;cStoolXpert;cOutpt_PHC;cTBTreat)] \ effSurvive
Death from TB
[sum(cOutpt_PHC;cHIV;cLAM;cStoolXpert;cOutpt_PHC;cTBTreat)] \ effDeath
Score >10
Initiate tx
Death, other causes
[sum(cOutpt_PHC;cHIV;cLAM;cStoolXpert;cOutpt_PHC;cTBTreat)] \ effDeath
Self-cure
[sum(cOutpt_PHC;cHIV;cLAM;cStoolXpert;cOutpt_PHC;cTBTreat*0.5)] \ effSurvive
LTFU, partial Tx
Death from TB
[sum(cOutpt_PHC;cHIV;cLAM;cStoolXpert;cOutpt_PHC;cTBTreat*0.5)] \ effDeath
Stay at PHC
Death, other causes
[sum(cOutpt_PHC;cHIV;cLAM;cStoolXpert;cOutpt_PHC;cTBTreat*0.5)] \ effDeath
Urine LAM negative
Death from TB
[sum(cOutpt_PHC;cHIV;cLAM;cStoolXpert;cOutpt_PHC)] \ effDeath
Score <10
Follow-up
Self-cure
[sum(cOutpt_PHC;cHIV;cLAM;cStoolXpert;cOutpt_PHC)] \ effSurvive
Death, non-TB
[sum(cOutpt_PHC;cHIV;cLAM;cStoolXpert;cOutpt_PHC)] \ effDeath
Complete Tx
[sum(cOutpt_PHC;cHIV;cLAM;cCXR;cSputumXpert;cTBTreat)] \ effSurvive
Death from TB
[sum(cOutpt_PHC;cHIV;cLAM;cCXR;cSputumXpert;cTBTreat)] \ effDeath
Sputum Xpert-positive
Initiate Tx
Death, other causes
[sum(cOutpt_PHC;cHIV;cLAM;cCXR;cSputumXpert;cTBTreat)] \ effDeath
Self-cure
[sum(cOutpt_PHC;cHIV;cLAM;cCXR;cSputumXpert;cTBTreat*0.5)] \ effSurvive
LTFU, partial Tx
Death from TB
[sum(cOutpt_PHC;cHIV;cLAM;cCXR;cSputumXpert;cTBTreat*0.5)] \ effDeath
Death, other causes
[sum(cOutpt_PHC;cHIV;cLAM;cCXR;cSputumXpert;cTBTreat*0.5)] \ effDeath
Referral to DH
Complete Tx
[sum(cOutpt_PHC;cHIV;cLAM;cCXR;cSputumXpert;cOutpt_DH;cTBTreat)] \ effSurvive
Death from TB
[sum(cOutpt_PHC;cHIV;cLAM;cCXR;cSputumXpert;cOutpt_DH;cTBTreat)] \ effDeath
Score w CXR >10
Initiate tx
Death, other causes
[sum(cOutpt_PHC;cHIV;cLAM;cCXR;cSputumXpert;cOutpt_DH;cTBTreat)] \ effDeath
Self-cure
[sum(cOutpt_PHC;cHIV;cLAM;cCXR;cSputumXpert;cOutpt_DH;cTBTreat*0.5)] \ effSurvive
LTFU, partial Tx
Death from TB
[sum(cOutpt_PHC;cHIV;cLAM;cCXR;cSputumXpert;cOutpt_DH;cTBTreat*0.5)] \ effDeath
Sputum Xpert-negative
Death, other causes
[sum(cOutpt_PHC;cHIV;cLAM;cCXR;cSputumXpert;cOutpt_DH;cTBTreat*0.5)] \ effDeath
Death from TB
[sum(cOutpt_PHC;cHIV;cLAM;cCXR;cSputumXpert;cOutpt_DH)] \ effDeath
Stool - no
Score w CXR <10
Follow-up
Self-cure
[sum(cOutpt_PHC;cHIV;cLAM;cCXR;cSputumXpert;cOutpt_DH)] \ effSurvive
Death, non-TB
[sum(cOutpt_PHC;cHIV;cLAM;cCXR;cSputumXpert;cOutpt_DH)] \ effDeath
Complete Tx
High risk, HIV+
[sum(cOutpt_PHC;cHIV;cLAM;cOutpt_PHC;cTBTreat)] \ effSurvive
Death from TB
[sum(cOutpt_PHC;cHIV;cLAM;cOutpt_PHC;cTBTreat)] \ effDeath
Score >10
Initiate tx
Death, other causes
[sum(cOutpt_PHC;cHIV;cLAM;cOutpt_PHC;cTBTreat)] \ effDeath
Self-cure
[sum(cOutpt_PHC;cHIV;cLAM;cOutpt_PHC;cTBTreat*0.5)] \ effSurvive
LTFU, partial Tx
Death from TB
[sum(cOutpt_PHC;cHIV;cLAM;cOutpt_PHC;cTBTreat*0.5)] \ effDeath
Stay at PHC
Death, other causes
[sum(cOutpt_PHC;cHIV;cLAM;cOutpt_PHC;cTBTreat*0.5)] \ effDeath
Death from TB
[sum(cOutpt_PHC;cHIV;cLAM;cOutpt_PHC)] \ effDeath
Score <10
Follow-up
Self-cure
[sum(cOutpt_PHC;cHIV;cLAM;cOutpt_PHC)] \ effSurvive
Death, non-TB
[sum(cOutpt_PHC;cHIV;cLAM;cOutpt_PHC)] \ effDeath
Complete Tx
[sum(cOutpt_PHC;cHIV;cStoolXpert;cTBTreat)] \ effSurvive
Death from TB
[sum(cOutpt_PHC;cHIV;cStoolXpert;cTBTreat)] \ effDeath
Stool Xpert-positive
Inititate Tx
Death, other causes
[sum(cOutpt_PHC;cHIV;cStoolXpert;cTBTreat)] \ effDeath
Self-cure
[sum(cOutpt_PHC;cHIV;cStoolXpert;cTBTreat*0.5)] \ effSurvive
LTFU, partial Tx
Death from TB
[sum(cOutpt_PHC;cHIV;cStoolXpert;cTBTreat*0.5)] \ effDeath
Death, other causes
[sum(cOutpt_PHC;cHIV;cStoolXpert;cTBTreat*0.5)] \ effDeath
Complete Tx
[sum(cOutpt_PHC;cHIV;cStoolXpert;cCXR;cSputumXpert;cTBTreat)] \ effSurvive
Death from TB
[sum(cOutpt_PHC;cHIV;cStoolXpert;cCXR;cSputumXpert;cTBTreat)] \ effDeath
Sputum Xpert-positive
Initiate Tx
Death, other causes
[sum(cOutpt_PHC;cHIV;cStoolXpert;cCXR;cSputumXpert;cTBTreat)] \ effDeath
Self-cure
[sum(cOutpt_PHC;cHIV;cStoolXpert;cCXR;cSputumXpert;cTBTreat*0.5)] \ effSurvive
LTFU, partial Tx
Death from TB
[sum(cOutpt_PHC;cHIV;cStoolXpert;cCXR;cSputumXpert;cTBTreat*0.5)] \ effDeath
Stool - yes
Death, other causes
[sum(cOutpt_PHC;cHIV;cStoolXpert;cCXR;cSputumXpert;cTBTreat*0.5)] \ effDeath
Complete Tx
Referral to DH
[sum(cOutpt_PHC;cHIV;cStoolXpert;cCXR;cSputumXpert;cOutpt_DH;cTBTreat)] \ effSurvive
Death from TB
[sum(cOutpt_PHC;cHIV;cStoolXpert;cCXR;cSputumXpert;cOutpt_DH;cTBTreat)] \ effDeath
Score w CXR >10
Initiate tx
Death, other causes
[sum(cOutpt_PHC;cHIV;cStoolXpert;cCXR;cSputumXpert;cOutpt_DH;cTBTreat)] \ effDeath
Self-cure
[sum(cOutpt_PHC;cHIV;cStoolXpert;cCXR;cSputumXpert;cOutpt_DH;cTBTreat*0.5)] \ effSurvive
LTFU, partial Tx
Death from TB
[sum(cOutpt_PHC;cHIV;cStoolXpert;cCXR;cSputumXpert;cOutpt_DH;cTBTreat*0.5)] \ effDeath
Sputum Xpert-negative
Death, other causes
[sum(cOutpt_PHC;cHIV;cStoolXpert;cCXR;cSputumXpert;cOutpt_DH;cTBTreat*0.5)] \ effDeath
Death from TB
[sum(cOutpt_PHC;cHIV;cStoolXpert;cCXR;cSputumXpert;cOutpt_DH)] \ effDeath
Stool Xpert-negative
Score w CXR <10
Follow-up
Self-cure
[sum(cOutpt_PHC;cHIV;cStoolXpert;cCXR;cSputumXpert;cOutpt_DH)] \ effSurvive
Death, non-TB
[sum(cOutpt_PHC;cHIV;cStoolXpert;cCXR;cSputumXpert;cOutpt_DH)] \ effDeath
Complete Tx
[sum(cOutpt_PHC;cHIV;cStoolXpert;cOutpt_PHC;cTBTreat)] \ effSurvive
Death from TB
[sum(cOutpt_PHC;cHIV;cStoolXpert;cOutpt_PHC;cTBTreat)] \ effDeath
Score >10
Initiate tx
Death, other causes
[sum(cOutpt_PHC;cHIV;cStoolXpert;cOutpt_PHC;cTBTreat)] \ effDeath
Self-cure
[sum(cOutpt_PHC;cHIV;cStoolXpert;cOutpt_PHC;cTBTreat*0.5)] \ effSurvive
Urine - no
LTFU, partial Tx
Stay at PHC
Death from TB
[sum(cOutpt_PHC;cHIV;cStoolXpert;cOutpt_PHC;cTBTreat*0.5)] \ effDeath
Death, other causes
[sum(cOutpt_PHC;cHIV;cStoolXpert;cOutpt_PHC;cTBTreat*0.5)] \ effDeath
Death from TB
[sum(cOutpt_PHC;cHIV;cStoolXpert;cOutpt_PHC)] \ effDeath
Score <10
Follow-up
Self-cure
[sum(cOutpt_PHC;cHIV;cStoolXpert;cOutpt_PHC)] \ effSurvive
Death, non-TB
[sum(cOutpt_PHC;cHIV;cStoolXpert;cOutpt_PHC)] \ effDeath
Complete Tx
[sum(cOutpt_PHC;cHIV;cOutpt_PHC;cTBTreat)] \ effSurvive
Death from TB
[sum(cOutpt_PHC;cHIV;cOutpt_PHC;cTBTreat)] \ effDeath
Score >10
Initiate tx
Death, other causes
[sum(cOutpt_PHC;cHIV;cOutpt_PHC;cTBTreat)] \ effDeath
Self-cure
[sum(cOutpt_PHC;cHIV;cOutpt_PHC;cTBTreat*0.5)] \ effSurvive
LTFU, partial Tx
Death from TB
[sum(cOutpt_PHC;cHIV;cOutpt_PHC;cTBTreat*0.5)] \ effDeath
Stay at PHC
Death, other causes
[sum(cOutpt_PHC;cHIV;cOutpt_PHC;cTBTreat*0.5)] \ effDeath
Death from TB
[sum(cOutpt_PHC;cHIV;cOutpt_PHC)] \ effDeath
Score <10
Follow-up
Self-cure
[sum(cOutpt_PHC;cHIV;cOutpt_PHC)] \ effSurvive
Death, non-TB
[sum(cOutpt_PHC;cHIV;cOutpt_PHC)] \ effDeath
Complete Tx
[sum(cOutpt_PHC;cHIV;cOutpt_PHC;cCXR;cSputumXpert;cTBTreat)] \ effSurvive
Stool - no
Death from TB
[sum(cOutpt_PHC;cHIV;cOutpt_PHC;cCXR;cSputumXpert;cTBTreat)] \ effDeath
Sputum Xpert-positive
Initiate Tx
Death, other causes
[sum(cOutpt_PHC;cHIV;cOutpt_PHC;cCXR;cSputumXpert;cTBTreat)] \ effDeath
Self-cure
[sum(cOutpt_PHC;cHIV;cOutpt_PHC;cCXR;cSputumXpert;cTBTreat*0.5)] \ effSurvive
LTFU, partial Tx
Death from TB
[sum(cOutpt_PHC;cHIV;cOutpt_PHC;cCXR;cSputumXpert;cTBTreat*0.5)] \ effDeath
Death, other causes
[sum(cOutpt_PHC;cHIV;cOutpt_PHC;cCXR;cSputumXpert;cTBTreat*0.5)] \ effDeath
Referral to DH
Complete Tx
[sum(cOutpt_PHC;cHIV;cOutpt_PHC;cCXR;cSputumXpert;cOutpt_DH;cTBTreat)] \ effSurvive
Death from TB
[sum(cOutpt_PHC;cHIV;cOutpt_PHC;cCXR;cSputumXpert;cOutpt_DH;cTBTreat)] \ effDeath
Score w CXR >10
Initiate tx
Death, other causes
[sum(cOutpt_PHC;cHIV;cOutpt_PHC;cCXR;cSputumXpert;cOutpt_DH;cTBTreat)] \ effDeath
Self-cure
[sum(cOutpt_PHC;cHIV;cOutpt_PHC;cCXR;cSputumXpert;cOutpt_DH;cTBTreat*0.5)] \ effSurvive
Child <5 with presumptive TB
LTFU, partial Tx
Death from TB
[sum(cOutpt_PHC;cHIV;cOutpt_PHC;cCXR;cSputumXpert;cOutpt_DH;cTBTreat*0.5)] \ effDeath
Sputum Xpert-negative
Death, other causes
[sum(cOutpt_PHC;cHIV;cOutpt_PHC;cCXR;cSputumXpert;cOutpt_DH;cTBTreat*0.5)] \ effDeath
Death from TB
[sum(cOutpt_PHC;cHIV;cOutpt_PHC;cCXR;cSputumXpert;cOutpt_DH)] \ effDeath
Score w CXR <10
Follow-up
Self-cure
[sum(cOutpt_PHC;cHIV;cOutpt_PHC;cCXR;cSputumXpert;cOutpt_DH)] \ effSurvive
Death, non-TB
[sum(cOutpt_PHC;cHIV;cOutpt_PHC;cCXR;cSputumXpert;cOutpt_DH)] \ effDeath

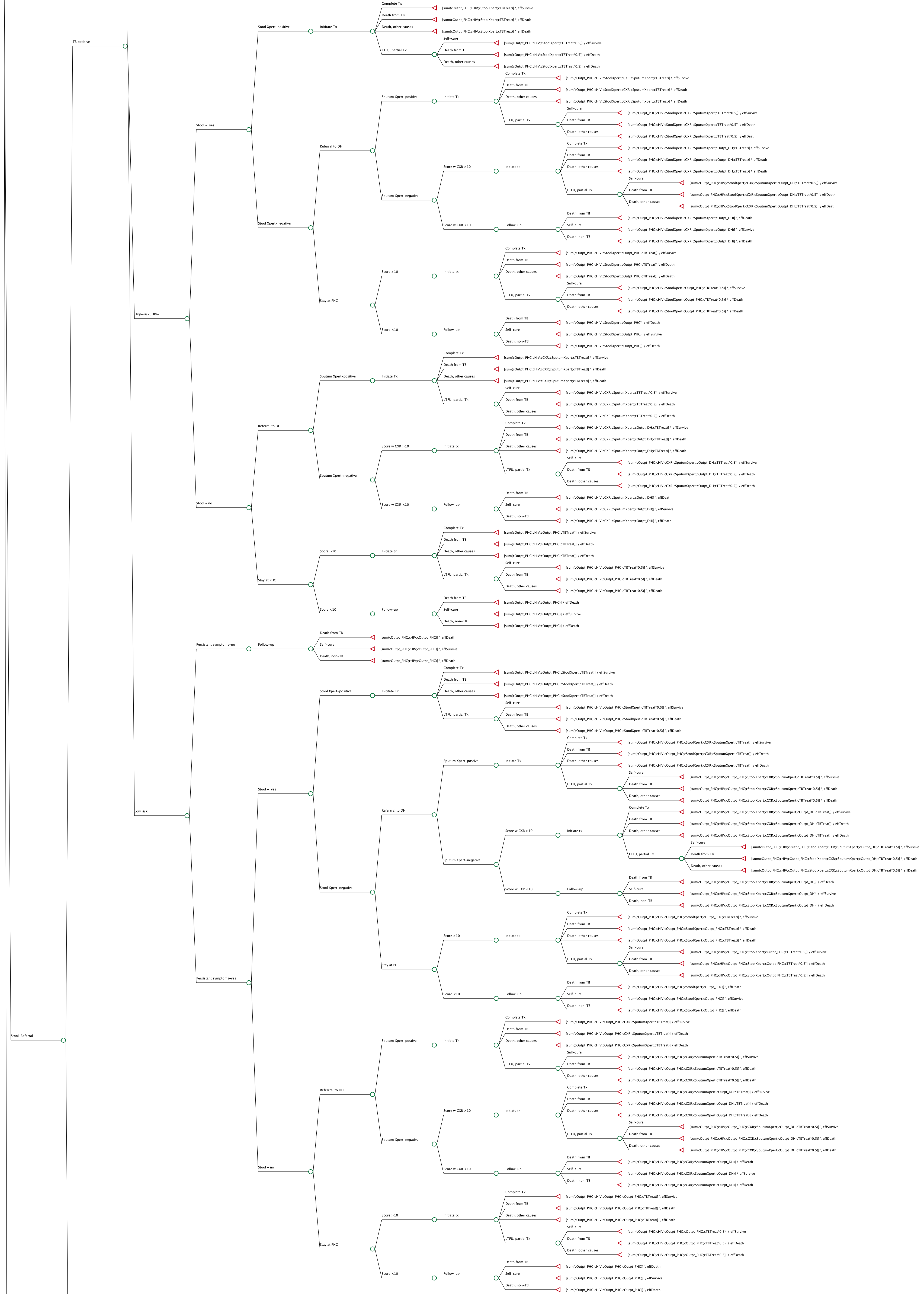

TB positive
Stool Xpert–positive
Inititate Tx
Complete Tx
[sum(cOutpt_PHC;cHIV;cStoolXpert;cTBTreat)] \ effSurvive
Death from TB
[sum(cOutpt_PHC;cHIV;cStoolXpert;cTBTreat)] \ effDeath
Death, other causes
[sum(cOutpt_PHC;cHIV;cStoolXpert;cTBTreat)] \ effDeath
LTFU, partial Tx
Self–cure
[sum(cOutpt_PHC;cHIV;cStoolXpert;cTBTreat*0.5)] \ effSurvive
Death from TB
[sum(cOutpt_PHC;cHIV;cStoolXpert;cTBTreat*0.5)] \ effDeath
Death, other causes
[sum(cOutpt_PHC;cHIV;cStoolXpert;cTBTreat*0.5)] \ effDeath
High–risk, HIV–
Stool – yes
Stool Xpert–negative
Referral to DH
Sputum Xpert–positive
Initiate Tx
Complete Tx
[sum(cOutpt_PHC;cHIV;cStoolXpert;cCXR;cSputumXpert;cTBTreat)] \ effSurvive
Death from TB
[sum(cOutpt_PHC;cHIV;cStoolXpert;cCXR;cSputumXpert;cTBTreat)] \ effDeath
Death, other causes
[sum(cOutpt_PHC;cHIV;cStoolXpert;cCXR;cSputumXpert;cTBTreat)] \ effDeath
LTFU, partial Tx
Self–cure
[sum(cOutpt_PHC;cHIV;cStoolXpert;cCXR;cSputumXpert;cTBTreat*0.5)] \ effSurvive
Death from TB
[sum(cOutpt_PHC;cHIV;cStoolXpert;cCXR;cSputumXpert;cTBTreat*0.5)] \ effDeath
Death, other causes
[sum(cOutpt_PHC;cHIV;cStoolXpert;cCXR;cSputumXpert;cTBTreat*0.5)] \ effDeath
Sputum Xpert–negative
Score w CXR >10
Initiate tx
Complete Tx
[sum(cOutpt_PHC;cHIV;cStoolXpert;cCXR;cSputumXpert;cOutpt_DH;cTBTreat)] \ effSurvive
Death from TB
[sum(cOutpt_PHC;cHIV;cStoolXpert;cCXR;cSputumXpert;cOutpt_DH;cTBTreat)] \ effDeath
Death, other causes
[sum(cOutpt_PHC;cHIV;cStoolXpert;cCXR;cSputumXpert;cOutpt_DH;cTBTreat)] \ effDeath
LTFU, partial Tx
Self–cure
[sum(cOutpt_PHC;cHIV;cStoolXpert;cCXR;cSputumXpert;cOutpt_DH;cTBTreat*0.5)] \ effSurvive
Death from TB
[sum(cOutpt_PHC;cHIV;cStoolXpert;cCXR;cSputumXpert;cOutpt_DH;cTBTreat*0.5)] \ effDeath
Death, other causes
[sum(cOutpt_PHC;cHIV;cStoolXpert;cCXR;cSputumXpert;cOutpt_DH;cTBTreat*0.5)] \ effDeath
Score w CXR <10
Follow–up
Death from TB
[sum(cOutpt_PHC;cHIV;cStoolXpert;cCXR;cSputumXpert;cOutpt_DH)] \ effDeath
Self–cure
[sum(cOutpt_PHC;cHIV;cStoolXpert;cCXR;cSputumXpert;cOutpt_DH)] \ effSurvive
Death, non–TB
[sum(cOutpt_PHC;cHIV;cStoolXpert;cCXR;cSputumXpert;cOutpt_DH)] \ effDeath
Stay at PHC
Score >10
Initiate tx
Complete Tx
[sum(cOutpt_PHC;cHIV;cStoolXpert;cOutpt_PHC;cTBTreat)] \ effSurvive
Death from TB
[sum(cOutpt_PHC;cHIV;cStoolXpert;cOutpt_PHC;cTBTreat)] \ effDeath
Death, other causes
[sum(cOutpt_PHC;cHIV;cStoolXpert;cOutpt_PHC;cTBTreat)] \ effDeath
LTFU, partial Tx
Self–cure
[sum(cOutpt_PHC;cHIV;cStoolXpert;cOutpt_PHC;cTBTreat*0.5)] \ effSurvive
Death from TB
[sum(cOutpt_PHC;cHIV;cStoolXpert;cOutpt_PHC;cTBTreat*0.5)] \ effDeath
Death, other causes
[sum(cOutpt_PHC;cHIV;cStoolXpert;cOutpt_PHC;cTBTreat*0.5)] \ effDeath
Score <10
Follow–up
Death from TB
[sum(cOutpt_PHC;cHIV;cStoolXpert;cOutpt_PHC)] \ effDeath
Self–cure
[sum(cOutpt_PHC;cHIV;cStoolXpert;cOutpt_PHC)] \ effSurvive
Death, non–TB
[sum(cOutpt_PHC;cHIV;cStoolXpert;cOutpt_PHC)] \ effDeath
Stool – no
Referral to DH
Sputum Xpert–positive
Initiate Tx
Complete Tx
[sum(cOutpt_PHC;cHIV;cCXR;cSputumXpert;cTBTreat)] \ effSurvive
Death from TB
[sum(cOutpt_PHC;cHIV;cCXR;cSputumXpert;cTBTreat)] \ effDeath
Death, other causes
[sum(cOutpt_PHC;cHIV;cCXR;cSputumXpert;cTBTreat)] \ effDeath
LTFU, partial Tx
Self–cure
[sum(cOutpt_PHC;cHIV;cCXR;cSputumXpert;cTBTreat*0.5)] \ effSurvive
Death from TB
[sum(cOutpt_PHC;cHIV;cCXR;cSputumXpert;cTBTreat*0.5)] \ effDeath
Death, other causes
[sum(cOutpt_PHC;cHIV;cCXR;cSputumXpert;cTBTreat*0.5)] \ effDeath
Sputum Xpert–negative
Score w CXR >10
Initiate tx
Complete Tx
[sum(cOutpt_PHC;cHIV;cCXR;cSputumXpert;cOutpt_DH;cTBTreat)] \ effSurvive
Death from TB
[sum(cOutpt_PHC;cHIV;cCXR;cSputumXpert;cOutpt_DH;cTBTreat)] \ effDeath
Death, other causes
[sum(cOutpt_PHC;cHIV;cCXR;cSputumXpert;cOutpt_DH;cTBTreat)] \ effDeath
LTFU, partial Tx
Self–cure
[sum(cOutpt_PHC;cHIV;cCXR;cSputumXpert;cOutpt_DH;cTBTreat*0.5)] \ effSurvive
Death from TB
[sum(cOutpt_PHC;cHIV;cCXR;cSputumXpert;cOutpt_DH;cTBTreat*0.5)] \ effDeath
Death, other causes
[sum(cOutpt_PHC;cHIV;cCXR;cSputumXpert;cOutpt_DH;cTBTreat*0.5)] \ effDeath
Score w CXR <10
Follow–up
Death from TB
[sum(cOutpt_PHC;cHIV;cCXR;cSputumXpert;cOutpt_DH)] \ effDeath
Self–cure
[sum(cOutpt_PHC;cHIV;cCXR;cSputumXpert;cOutpt_DH)] \ effSurvive
Death, non–TB
[sum(cOutpt_PHC;cHIV;cCXR;cSputumXpert;cOutpt_DH)] \ effDeath
Stay at PHC
Score >10
Initiate tx
Complete Tx
[sum(cOutpt_PHC;cHIV;cOutpt_PHC;cTBTreat)] \ effSurvive
Death from TB
[sum(cOutpt_PHC;cHIV;cOutpt_PHC;cTBTreat)] \ effDeath
Death, other causes
[sum(cOutpt_PHC;cHIV;cOutpt_PHC;cTBTreat)] \ effDeath
LTFU, partial Tx
Self–cure
[sum(cOutpt_PHC;cHIV;cOutpt_PHC;cTBTreat*0.5)] \ effSurvive
Death from TB
[sum(cOutpt_PHC;cHIV;cOutpt_PHC;cTBTreat*0.5)] \ effDeath
Death, other causes
[sum(cOutpt_PHC;cHIV;cOutpt_PHC;cTBTreat*0.5)] \ effDeath
Score <10
Follow–up
Death from TB
[sum(cOutpt_PHC;cHIV;cOutpt_PHC)] \ effDeath
Self–cure
[sum(cOutpt_PHC;cHIV;cOutpt_PHC)] \ effSurvive
Death, non–TB
[sum(cOutpt_PHC;cHIV;cOutpt_PHC)] \ effDeath
Low risk
Persistent symptoms–no
Follow–up
Death from TB
[sum(cOutpt_PHC;cHIV;cOutpt_PHC)] \ effDeath
Self–cure
[sum(cOutpt_PHC;cHIV;cOutpt_PHC)] \ effSurvive
Death, non–TB
[sum(cOutpt_PHC;cHIV;cOutpt_PHC)] \ effDeath
Persistant symptoms–yes
Stool – yes
Stool Xpert–positive
Inititate Tx
Complete Tx
[sum(cOutpt_PHC;cHIV;cOutpt_PHC;cStoolXpert;cTBTreat)] \ effSurvive
Death from TB
[sum(cOutpt_PHC;cHIV;cOutpt_PHC;cStoolXpert;cTBTreat)] \ effDeath
Death, other causes
[sum(cOutpt_PHC;cHIV;cOutpt_PHC;cStoolXpert;cTBTreat)] \ effDeath
LTFU, partial Tx
Self–cure
[sum(cOutpt_PHC;cHIV;cOutpt_PHC;cStoolXpert;cTBTreat*0.5)] \ effSurvive
Death from TB
[sum(cOutpt_PHC;cHIV;cOutpt_PHC;cStoolXpert;cTBTreat*0.5)] \ effDeath
Death, other causes
[sum(cOutpt_PHC;cHIV;cOutpt_PHC;cStoolXpert;cTBTreat*0.5)] \ effDeath
Stool Xpert–negative
Referrral to DH
Sputum Xpert–postive
Initiate Tx
Complete Tx
[sum(cOutpt_PHC;cHIV;cOutpt_PHC;cStoolXpert;cCXR;cSputumXpert;cTBTreat)] \ effSurvive
Death from TB
[sum(cOutpt_PHC;cHIV;cOutpt_PHC;cStoolXpert;cCXR;cSputumXpert;cTBTreat)] \ effDeath
Death, other causes
[sum(cOutpt_PHC;cHIV;cOutpt_PHC;cStoolXpert;cCXR;cSputumXpert;cTBTreat)] \ effDeath
LTFU, partial Tx
Self–cure
[sum(cOutpt_PHC;cHIV;cOutpt_PHC;cStoolXpert;cCXR;cSputumXpert;cTBTreat*0.5)] \ effSurvive
Death from TB
[sum(cOutpt_PHC;cHIV;cOutpt_PHC;cStoolXpert;cCXR;cSputumXpert;cTBTreat*0.5)] \ effDeath
Death, other causes
[sum(cOutpt_PHC;cHIV;cOutpt_PHC;cStoolXpert;cCXR;cSputumXpert;cTBTreat*0.5)] \ effDeath
Sputum Xpert–negative
Score w CXR >10
Initiate tx
Complete Tx
[sum(cOutpt_PHC;cHIV;cOutpt_PHC;cStoolXpert;cCXR;cSputumXpert;cOutpt_DH;cTBTreat)] \ effSurvive
Death from TB
[sum(cOutpt_PHC;cHIV;cOutpt_PHC;cStoolXpert;cCXR;cSputumXpert;cOutpt_DH;cTBTreat)] \ effDeath
Death, other causes
[sum(cOutpt_PHC;cHIV;cOutpt_PHC;cStoolXpert;cCXR;cSputumXpert;cOutpt_DH;cTBTreat)] \ effDeath
LTFU, partial Tx
Self–cure
[sum(cOutpt_PHC;cHIV;cOutpt_PHC;cStoolXpert;cCXR;cSputumXpert;cOutpt_DH;cTBTreat*0.5)] \ effSurvive
Death from TB
[sum(cOutpt_PHC;cHIV;cOutpt_PHC;cStoolXpert;cCXR;cSputumXpert;cOutpt_DH;cTBTreat*0.5)] \ effDeath
Death, other causes
[sum(cOutpt_PHC;cHIV;cOutpt_PHC;cStoolXpert;cCXR;cSputumXpert;cOutpt_DH;cTBTreat*0.5)] \ effDeath
Score w CXR <10
Follow–up
Death from TB
[sum(cOutpt_PHC;cHIV;cOutpt_PHC;cStoolXpert;cCXR;cSputumXpert;cOutpt_DH)] \ effDeath
Self–cure
[sum(cOutpt_PHC;cHIV;cOutpt_PHC;cStoolXpert;cCXR;cSputumXpert;cOutpt_DH)] \ effSurvive
Death, non–TB
[sum(cOutpt_PHC;cHIV;cOutpt_PHC;cStoolXpert;cCXR;cSputumXpert;cOutpt_DH)] \ effDeath
Stay at PHC
Score >10
Initiate tx
Complete Tx
[sum(cOutpt_PHC;cHIV;cOutpt_PHC;cStoolXpert;cOutpt_PHC;cTBTreat)] \ effSurvive
Death from TB
[sum(cOutpt_PHC;cHIV;cOutpt_PHC;cStoolXpert;cOutpt_PHC;cTBTreat)] \ effDeath
Death, other causes
[sum(cOutpt_PHC;cHIV;cOutpt_PHC;cStoolXpert;cOutpt_PHC;cTBTreat)] \ effDeath
LTFU, partial Tx
Self–cure
[sum(cOutpt_PHC;cHIV;cOutpt_PHC;cStoolXpert;cOutpt_PHC;cTBTreat*0.5)] \ effSurvive
Death from TB
[sum(cOutpt_PHC;cHIV;cOutpt_PHC;cStoolXpert;cOutpt_PHC;cTBTreat*0.5)] \ effDeath
Death, other causes
[sum(cOutpt_PHC;cHIV;cOutpt_PHC;cStoolXpert;cOutpt_PHC;cTBTreat*0.5)] \ effDeath
Score <10
Follow–up
Death from TB
[sum(cOutpt_PHC;cHIV;cOutpt_PHC;cStoolXpert;cOutpt_PHC)] \ effDeath
Self–cure
[sum(cOutpt_PHC;cHIV;cOutpt_PHC;cStoolXpert;cOutpt_PHC)] \ effSurvive
Death, non–TB
[sum(cOutpt_PHC;cHIV;cOutpt_PHC;cStoolXpert;cOutpt_PHC)] \ effDeath
Stool – no
Referrral to DH
Sputum Xpert–positive
Initiate Tx
Complete Tx
[sum(cOutpt_PHC;cHIV;cOutpt_PHC;cCXR;cSputumXpert;cTBTreat)] \ effSurvive
Death from TB
[sum(cOutpt_PHC;cHIV;cOutpt_PHC;cCXR;cSputumXpert;cTBTreat)] \ effDeath
Death, other causes
[sum(cOutpt_PHC;cHIV;cOutpt_PHC;cCXR;cSputumXpert;cTBTreat)] \ effDeath
LTFU, partial Tx
Self–cure
[sum(cOutpt_PHC;cHIV;cOutpt_PHC;cCXR;cSputumXpert;cTBTreat*0.5)] \ effSurvive
Death from TB
[sum(cOutpt_PHC;cHIV;cOutpt_PHC;cCXR;cSputumXpert;cTBTreat*0.5)] \ effDeath
Death, other causes
[sum(cOutpt_PHC;cHIV;cOutpt_PHC;cCXR;cSputumXpert;cTBTreat*0.5)] \ effDeath
Sputum Xpert–negative
Score w CXR >10
Initiate tx
Complete Tx
[sum(cOutpt_PHC;cHIV;cOutpt_PHC;cCXR;cSputumXpert;cOutpt_DH;cTBTreat)] \ effSurvive
Death from TB
[sum(cOutpt_PHC;cHIV;cOutpt_PHC;cCXR;cSputumXpert;cOutpt_DH;cTBTreat)] \ effDeath
Death, other causes
[sum(cOutpt_PHC;cHIV;cOutpt_PHC;cCXR;cSputumXpert;cOutpt_DH;cTBTreat)] \ effDeath
LTFU, partial Tx
Self–cure
[sum(cOutpt_PHC;cHIV;cOutpt_PHC;cCXR;cSputumXpert;cOutpt_DH;cTBTreat*0.5)] \ effSurvive
Death from TB
[sum(cOutpt_PHC;cHIV;cOutpt_PHC;cCXR;cSputumXpert;cOutpt_DH;cTBTreat*0.5)] \ effDeath
Death, other causes
[sum(cOutpt_PHC;cHIV;cOutpt_PHC;cCXR;cSputumXpert;cOutpt_DH;cTBTreat*0.5)] \ effDeath
Score w CXR <10
Follow–up
Death from TB
[sum(cOutpt_PHC;cHIV;cOutpt_PHC;cCXR;cSputumXpert;cOutpt_DH)] \ effDeath
Self–cure
[sum(cOutpt_PHC;cHIV;cOutpt_PHC;cCXR;cSputumXpert;cOutpt_DH)] \ effSurvive
Death, non–TB
[sum(cOutpt_PHC;cHIV;cOutpt_PHC;cCXR;cSputumXpert;cOutpt_DH)] \ effDeath
Stay at PHC
Score >10
Initiate tx
Complete Tx
[sum(cOutpt_PHC;cHIV;cOutpt_PHC;cOutpt_PHC;cTBTreat)] \ effSurvive
Death from TB
[sum(cOutpt_PHC;cHIV;cOutpt_PHC;cOutpt_PHC;cTBTreat)] \ effDeath
Death, other causes
[sum(cOutpt_PHC;cHIV;cOutpt_PHC;cOutpt_PHC;cTBTreat)] \ effDeath
LTFU, partial Tx
Self–cure
[sum(cOutpt_PHC;cHIV;cOutpt_PHC;cOutpt_PHC;cTBTreat*0.5)] \ effSurvive
Death from TB
[sum(cOutpt_PHC;cHIV;cOutpt_PHC;cOutpt_PHC;cTBTreat*0.5)] \ effDeath
Death, other causes
[sum(cOutpt_PHC;cHIV;cOutpt_PHC;cOutpt_PHC;cTBTreat*0.5)] \ effDeath
Score <10
Follow–up
Death from TB
[sum(cOutpt_PHC;cHIV;cOutpt_PHC;cOutpt_PHC)] \ effDeath
Self–cure
[sum(cOutpt_PHC;cHIV;cOutpt_PHC;cOutpt_PHC)] \ effSurvive
Death, non–TB
[sum(cOutpt_PHC;cHIV;cOutpt_PHC;cOutpt_PHC)] \ effDeath
Stool–Referral

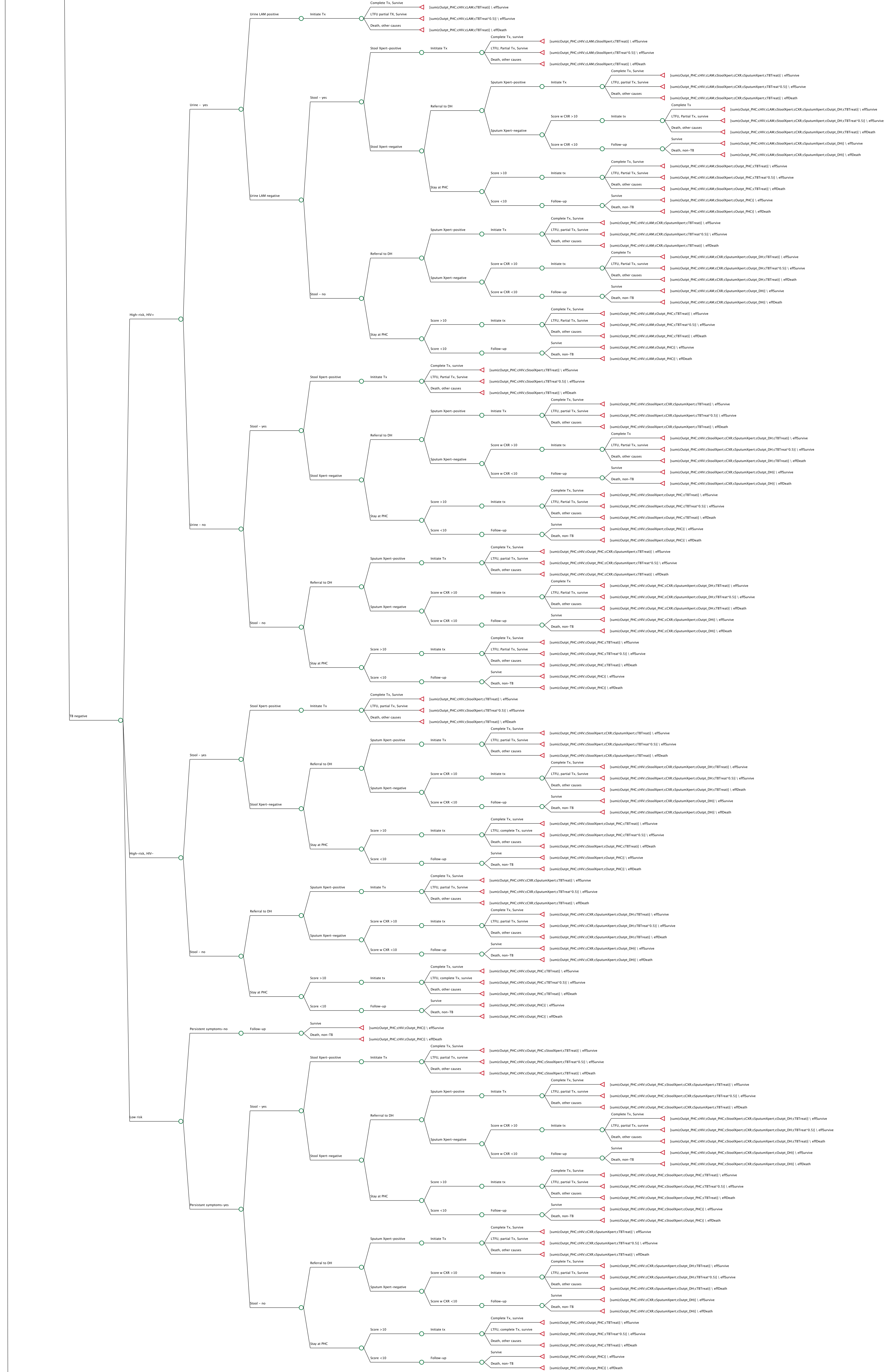

TB negative
High-risk, HIV+
Urine - yes
Urine LAM positive
Initiate Tx
Complete Tx, Survive
LTFU partial TX, Survive
Death, other causes
Urine LAM negative
Stool - yes
Stool Xpert-positive
Stool Xpert-negative
Referral to DH
Sputum Xpert-positive
Sputum Xpert-negative
Score w CXR >10
Score w CXR <10
Follow-up
Survive
Death, non-TB
Stay at PHC
Score >10
Score <10
Stool - no
Urine - no
High-risk, HIV-
Low risk
Persistent symptoms-no
Persistant symptoms-yes

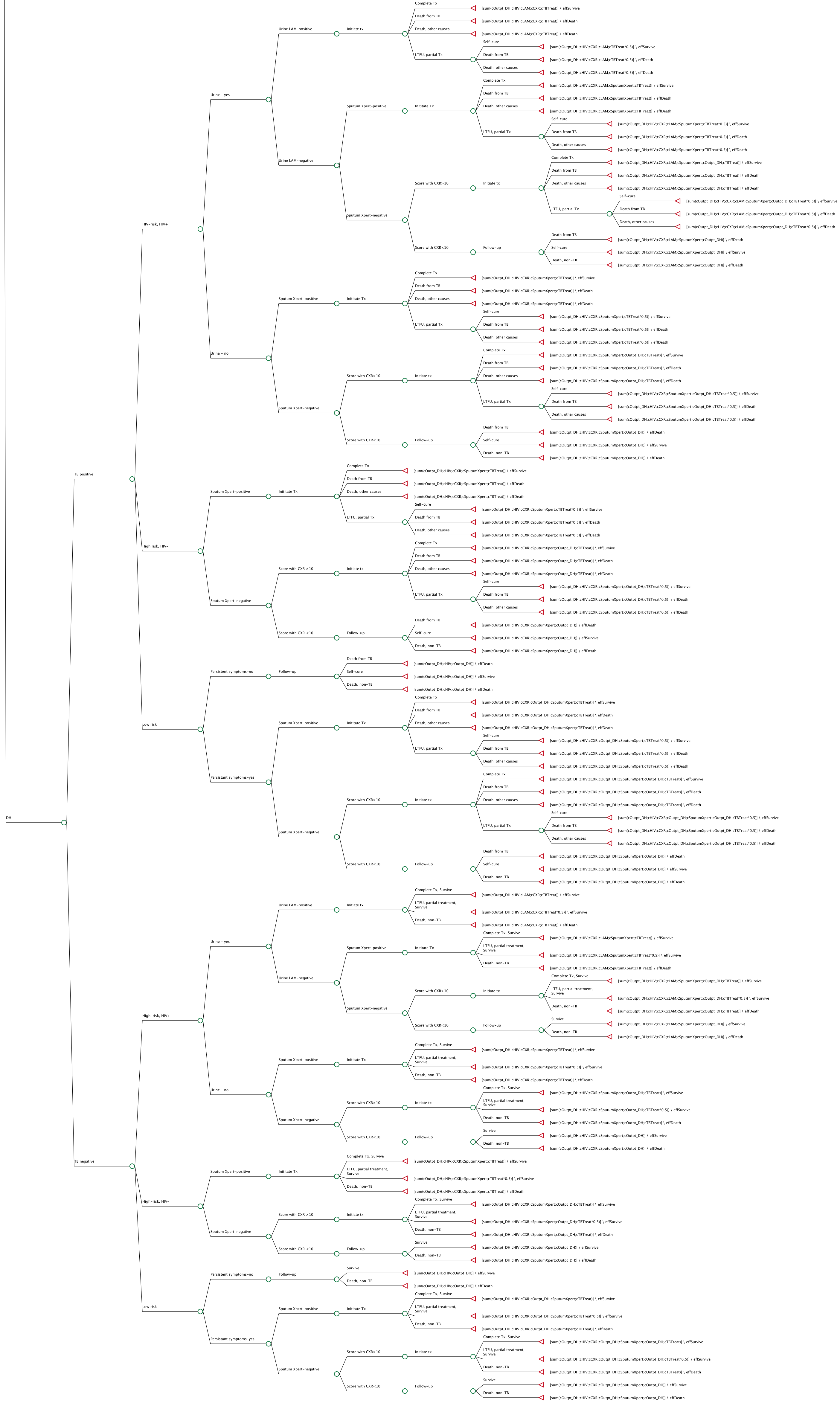

DH
TB positive
HIV−risk, HIV+
Urine − yes
Urine LAM−positive
Initiate tx
Complete Tx
[sum(cOutpt_DH;cHIV;cLAM;cCXR;cTBTreat)] \ effSurvive
Death from TB
[sum(cOutpt_DH;cHIV;cLAM;cCXR;cTBTreat)] \ effDeath
Death, other causes
[sum(cOutpt_DH;cHIV;cLAM;cCXR;cTBTreat)] \ effDeath
LTFU, partial Tx
Self−cure
[sum(cOutpt_DH;cHIV;cCXR;cLAM;cTBTreat*0.5)] \ effSurvive
Death from TB
[sum(cOutpt_DH;cHIV;cCXR;cLAM;cTBTreat*0.5)] \ effDeath
Death, other causes
[sum(cOutpt_DH;cHIV;cCXR;cLAM;cTBTreat*0.5)] \ effDeath
Urine LAM−negative
Sputum Xpert−positive
Initiate Tx
Complete Tx
[sum(cOutpt_DH;cHIV;cCXR;cLAM;cSputumXpert;cTBTreat)] \ effSurvive
Death from TB
[sum(cOutpt_DH;cHIV;cCXR;cLAM;cSputumXpert;cTBTreat)] \ effDeath
Death, other causes
[sum(cOutpt_DH;cHIV;cCXR;cLAM;cSputumXpert;cTBTreat)] \ effDeath
LTFU, partial Tx
Self−cure
[sum(cOutpt_DH;cHIV;cCXR;cLAM;cSputumXpert;cTBTreat*0.5)] \ effSurvive
Death from TB
[sum(cOutpt_DH;cHIV;cCXR;cLAM;cSputumXpert;cTBTreat*0.5)] \ effDeath
Death, other causes
[sum(cOutpt_DH;cHIV;cCXR;cLAM;cSputumXpert;cTBTreat*0.5)] \ effDeath
Sputum Xpert−negative
Score with CXR>10
Initiate tx
Complete Tx
[sum(cOutpt_DH;cHIV;cCXR;cLAM;cSputumXpert;cOutpt_DH;cTBTreat)] \ effSurvive
Death from TB
[sum(cOutpt_DH;cHIV;cCXR;cLAM;cSputumXpert;cOutpt_DH;cTBTreat)] \ effDeath
Death, other causes
[sum(cOutpt_DH;cHIV;cCXR;cLAM;cSputumXpert;cOutpt_DH;cTBTreat)] \ effDeath
LTFU, partial Tx
Self−cure
[sum(cOutpt_DH;cHIV;cCXR;cLAM;cSputumXpert;cOutpt_DH;cTBTreat*0.5)] \ effSurvive
Death from TB
[sum(cOutpt_DH;cHIV;cCXR;cLAM;cSputumXpert;cOutpt_DH;cTBTreat*0.5)] \ effDeath
Death, other causes
[sum(cOutpt_DH;cHIV;cCXR;cLAM;cSputumXpert;cOutpt_DH;cTBTreat*0.5)] \ effDeath
Score with CXR<10
Follow−up
Death from TB
[sum(cOutpt_DH;cHIV;cCXR;cLAM;cSputumXpert;cOutpt_DH)] \ effDeath
Self−cure
[sum(cOutpt_DH;cHIV;cCXR;cLAM;cSputumXpert;cOutpt_DH)] \ effSurvive
Death, non−TB
[sum(cOutpt_DH;cHIV;cCXR;cLAM;cSputumXpert;cOutpt_DH)] \ effDeath
Urine − no
Sputum Xpert−positive
Initiate Tx
Complete Tx
[sum(cOutpt_DH;cHIV;cCXR;cSputumXpert;cTBTreat)] \ effSurvive
Death from TB
[sum(cOutpt_DH;cHIV;cCXR;cSputumXpert;cTBTreat)] \ effDeath
Death, other causes
[sum(cOutpt_DH;cHIV;cCXR;cSputumXpert;cTBTreat)] \ effDeath
LTFU, partial Tx
Self−cure
[sum(cOutpt_DH;cHIV;cCXR;cSputumXpert;cTBTreat*0.5)] \ effSurvive
Death from TB
[sum(cOutpt_DH;cHIV;cCXR;cSputumXpert;cTBTreat*0.5)] \ effDeath
Death, other causes
[sum(cOutpt_DH;cHIV;cCXR;cSputumXpert;cTBTreat*0.5)] \ effDeath
Sputum Xpert−negative
Score with CXR>10
Initiate tx
Complete Tx
[sum(cOutpt_DH;cHIV;cCXR;cSputumXpert;cOutpt_DH;cTBTreat)] \ effSurvive
Death from TB
[sum(cOutpt_DH;cHIV;cCXR;cSputumXpert;cOutpt_DH;cTBTreat)] \ effDeath
Death, other causes
[sum(cOutpt_DH;cHIV;cCXR;cSputumXpert;cOutpt_DH;cTBTreat)] \ effDeath
LTFU, partial Tx
Self−cure
[sum(cOutpt_DH;cHIV;cCXR;cSputumXpert;cOutpt_DH;cTBTreat*0.5)] \ effSurvive
Death from TB
[sum(cOutpt_DH;cHIV;cCXR;cSputumXpert;cOutpt_DH;cTBTreat*0.5)] \ effDeath
Death, other causes
[sum(cOutpt_DH;cHIV;cCXR;cSputumXpert;cOutpt_DH;cTBTreat*0.5)] \ effDeath
Score with CXR<10
Follow−up
Death from TB
[sum(cOutpt_DH;cHIV;cCXR;cSputumXpert;cOutpt_DH)] \ effDeath
Self−cure
[sum(cOutpt_DH;cHIV;cCXR;cSputumXpert;cOutpt_DH)] \ effSurvive
Death, non−TB
[sum(cOutpt_DH;cHIV;cCXR;cSputumXpert;cOutpt_DH)] \ effDeath
High risk, HIV−
Sputum Xpert−positive
Inititate Tx
Complete Tx
[sum(cOutpt_DH;cHIV;cCXR;cSputumXpert;cTBTreat)] \ effSurvive
Death from TB
[sum(cOutpt_DH;cHIV;cCXR;cSputumXpert;cTBTreat)] \ effDeath
Death, other causes
[sum(cOutpt_DH;cHIV;cCXR;cSputumXpert;cTBTreat)] \ effDeath
LTFU, partial Tx
Self−cure
[sum(cOutpt_DH;cHIV;cCXR;cSputumXpert;cTBTreat*0.5)] \ effSurvive
Death from TB
[sum(cOutpt_DH;cHIV;cCXR;cSputumXpert;cTBTreat*0.5)] \ effDeath
Death, other causes
[sum(cOutpt_DH;cHIV;cCXR;cSputumXpert;cTBTreat*0.5)] \ effDeath
Sputum Xpert−negative
Score with CXR >10
Initiate tx
Complete Tx
[sum(cOutpt_DH;cHIV;cCXR;cSputumXpert;cOutpt_DH;cTBTreat)] \ effSurvive
Death from TB
[sum(cOutpt_DH;cHIV;cCXR;cSputumXpert;cOutpt_DH;cTBTreat)] \ effDeath
Death, other causes
[sum(cOutpt_DH;cHIV;cCXR;cSputumXpert;cOutpt_DH;cTBTreat)] \ effDeath
LTFU, partial Tx
Self−cure
[sum(cOutpt_DH;cHIV;cCXR;cSputumXpert;cOutpt_DH;cTBTreat*0.5)] \ effSurvive
Death from TB
[sum(cOutpt_DH;cHIV;cCXR;cSputumXpert;cOutpt_DH;cTBTreat*0.5)] \ effDeath
Death, other causes
[sum(cOutpt_DH;cHIV;cCXR;cSputumXpert;cOutpt_DH;cTBTreat*0.5)] \ effDeath
Score with CXR <10
Follow−up
Death from TB
[sum(cOutpt_DH;cHIV;cCXR;cSputumXpert;cOutpt_DH)] \ effDeath
Self−cure
[sum(cOutpt_DH;cHIV;cCXR;cSputumXpert;cOutpt_DH)] \ effSurvive
Death, non−TB
[sum(cOutpt_DH;cHIV;cCXR;cSputumXpert;cOutpt_DH)] \ effDeath
Low risk
Persistent symptoms−no
Follow−up
Death from TB
[sum(cOutpt_DH;cHIV;cOutpt_DH)] \ effDeath
Self−cure
[sum(cOutpt_DH;cHIV;cOutpt_DH)] \ effSurvive
Death, non−TB
[sum(cOutpt_DH;cHIV;cOutpt_DH)] \ effDeath
Persistant symptoms−yes
Sputum Xpert−positive
Initiate Tx
Complete Tx
[sum(cOutpt_DH;cHIV;cCXR;cOutpt_DH;cSputumXpert;cTBTreat)] \ effSurvive
Death from TB
[sum(cOutpt_DH;cHIV;cCXR;cOutpt_DH;cSputumXpert;cTBTreat)] \ effDeath
Death, other causes
[sum(cOutpt_DH;cHIV;cCXR;cOutpt_DH;cSputumXpert;cTBTreat)] \ effDeath
LTFU, partial Tx
Self−cure
[sum(cOutpt_DH;cHIV;cCXR;cOutpt_DH;cSputumXpert;cTBTreat*0.5)] \ effSurvive
Death from TB
[sum(cOutpt_DH;cHIV;cCXR;cOutpt_DH;cSputumXpert;cTBTreat*0.5)] \ effDeath
Death, other causes
[sum(cOutpt_DH;cHIV;cCXR;cOutpt_DH;cSputumXpert;cTBTreat*0.5)] \ effDeath
Sputum Xpert−negative
Score with CXR>10
Initiate tx
Complete Tx
[sum(cOutpt_DH;cHIV;cCXR;cOutpt_DH;cSputumXpert;cOutpt_DH;cTBTreat)] \ effSurvive
Death from TB
[sum(cOutpt_DH;cHIV;cCXR;cOutpt_DH;cSputumXpert;cOutpt_DH;cTBTreat)] \ effDeath
Death, other causes
[sum(cOutpt_DH;cHIV;cCXR;cOutpt_DH;cSputumXpert;cOutpt_DH;cTBTreat)] \ effDeath
LTFU, partial Tx
Self−cure
[sum(cOutpt_DH;cHIV;cCXR;cOutpt_DH;cSputumXpert;cOutpt_DH;cTBTreat*0.5)] \ effSurvive
Death from TB
[sum(cOutpt_DH;cHIV;cCXR;cOutpt_DH;cSputumXpert;cOutpt_DH;cTBTreat*0.5)] \ effDeath
Death, other causes
[sum(cOutpt_DH;cHIV;cCXR;cOutpt_DH;cSputumXpert;cOutpt_DH;cTBTreat*0.5)] \ effDeath
Score with CXR<10
Follow−up
Death from TB
[sum(cOutpt_DH;cHIV;cCXR;cOutpt_DH;cSputumXpert;cOutpt_DH)] \ effDeath
Self−cure
[sum(cOutpt_DH;cHIV;cCXR;cOutpt_DH;cSputumXpert;cOutpt_DH)] \ effSurvive
Death, non−TB
[sum(cOutpt_DH;cHIV;cCXR;cOutpt_DH;cSputumXpert;cOutpt_DH)] \ effDeath
TB negative
High−risk, HIV+
Urine − yes
Urine LAM−positive
Initiate tx
Complete Tx, Survive
[sum(cOutpt_DH;cHIV;cLAM;cCXR;cTBTreat)] \ effSurvive
LTFU, partial treatment, Survive
[sum(cOutpt_DH;cHIV;cLAM;cCXR;cTBTreat*0.5)] \ effSurvive
Death, non−TB
[sum(cOutpt_DH;cHIV;cLAM;cCXR;cTBTreat)] \ effDeath
Urine LAM−negative
Sputum Xpert−positive
Initiate Tx
Complete Tx, Survive
[sum(cOutpt_DH;cHIV;cCXR;cLAM;cSputumXpert;cTBTreat)] \ effSurvive
LTFU, partial treatment, Survive
[sum(cOutpt_DH;cHIV;cCXR;cLAM;cSputumXpert;cTBTreat*0.5)] \ effSurvive
Death, non−TB
[sum(cOutpt_DH;cHIV;cCXR;cLAM;cSputumXpert;cTBTreat)] \ effDeath
Sputum Xpert−negative
Score with CXR>10
Initiate tx
Complete Tx, Survive
[sum(cOutpt_DH;cHIV;cCXR;cLAM;cSputumXpert;cOutpt_DH;cTBTreat)] \ effSurvive
LTFU, partial treatment, Survive
[sum(cOutpt_DH;cHIV;cCXR;cLAM;cSputumXpert;cOutpt_DH;cTBTreat*0.5)] \ effSurvive
Death, non−TB
[sum(cOutpt_DH;cHIV;cCXR;cLAM;cSputumXpert;cOutpt_DH;cTBTreat)] \ effDeath
Score with CXR<10
Follow−up
Survive
[sum(cOutpt_DH;cHIV;cCXR;cLAM;cSputumXpert;cOutpt_DH)] \ effSurvive
Death, non−TB
[sum(cOutpt_DH;cHIV;cCXR;cLAM;cSputumXpert;cOutpt_DH)] \ effDeath
Urine − no
Sputum Xpert−positive
Initiate Tx
Complete Tx, Survive
[sum(cOutpt_DH;cHIV;cCXR;cSputumXpert;cTBTreat)] \ effSurvive
LTFU, partial treatment, Survive
[sum(cOutpt_DH;cHIV;cCXR;cSputumXpert;cTBTreat*0.5)] \ effSurvive
Death, non−TB
[sum(cOutpt_DH;cHIV;cCXR;cSputumXpert;cTBTreat)] \ effDeath
Sputum Xpert−negative
Score with CXR>10
Initiate tx
Complete Tx, Survive
[sum(cOutpt_DH;cHIV;cCXR;cSputumXpert;cOutpt_DH;cTBTreat)] \ effSurvive
LTFU, partial treatment, Survive
[sum(cOutpt_DH;cHIV;cCXR;cSputumXpert;cOutpt_DH;cTBTreat*0.5)] \ effSurvive
Death, non−TB
[sum(cOutpt_DH;cHIV;cCXR;cSputumXpert;cOutpt_DH;cTBTreat)] \ effDeath
Score with CXR<10
Follow−up
Survive
[sum(cOutpt_DH;cHIV;cCXR;cSputumXpert;cOutpt_DH)] \ effSurvive
Death, non−TB
[sum(cOutpt_DH;cHIV;cCXR;cSputumXpert;cOutpt_DH)] \ effDeath
High−risk, HIV−
Sputum Xpert−positive
Inititate Tx
Complete Tx, Survive
[sum(cOutpt_DH;cHIV;cCXR;cSputumXpert;cTBTreat)] \ effSurvive
LTFU, partial treatment, Survive
[sum(cOutpt_DH;cHIV;cCXR;cSputumXpert;cTBTreat*0.5)] \ effSurvive
Death, non−TB
[sum(cOutpt_DH;cHIV;cCXR;cSputumXpert;cTBTreat)] \ effDeath
Sputum Xpert−negative
Score with CXR >10
Initiate tx
Complete Tx, Survive
[sum(cOutpt_DH;cHIV;cCXR;cSputumXpert;cOutpt_DH;cTBTreat)] \ effSurvive
LTFU, partial treatment, Survive
[sum(cOutpt_DH;cHIV;cCXR;cSputumXpert;cOutpt_DH;cTBTreat*0.5)] \ effSurvive
Death, non−TB
[sum(cOutpt_DH;cHIV;cCXR;cSputumXpert;cOutpt_DH;cTBTreat)] \ effDeath
Score with CXR <10
Follow−up
Survive
[sum(cOutpt_DH;cHIV;cCXR;cSputumXpert;cOutpt_DH)] \ effSurvive
Death, non−TB
[sum(cOutpt_DH;cHIV;cCXR;cSputumXpert;cOutpt_DH)] \ effDeath
Low risk
Persistent symptoms−no
Follow−up
Survive
[sum(cOutpt_DH;cHIV;cOutpt_DH)] \ effSurvive
Death, non−TB
[sum(cOutpt_DH;cHIV;cOutpt_DH)] \ effDeath
Persistant symptoms−yes
Sputum Xpert−positive
Initiate Tx
Complete Tx, Survive
[sum(cOutpt_DH;cHIV;cCXR;cOutpt_DH;cSputumXpert;cTBTreat)] \ effSurvive
LTFU, partial treatment, Survive
[sum(cOutpt_DH;cHIV;cCXR;cOutpt_DH;cSputumXpert;cTBTreat*0.5)] \ effSurvive
Death, non−TB
[sum(cOutpt_DH;cHIV;cCXR;cOutpt_DH;cSputumXpert;cTBTreat)] \ effDeath
Sputum Xpert−negative
Score with CXR>10
Initiate tx
Complete Tx, Survive
[sum(cOutpt_DH;cHIV;cCXR;cOutpt_DH;cSputumXpert;cOutpt_DH;cTBTreat)] \ effSurvive
LTFU, partial treatment, Survive
[sum(cOutpt_DH;cHIV;cCXR;cOutpt_DH;cSputumXpert;cOutpt_DH;cTBTreat*0.5)] \ effSurvive
Death, non−TB
[sum(cOutpt_DH;cHIV;cCXR;cOutpt_DH;cSputumXpert;cOutpt_DH;cTBTreat)] \ effDeath
Score with CXR<10
Follow−up
Survive
[sum(cOutpt_DH;cHIV;cCXR;cOutpt_DH;cSputumXpert;cOutpt_DH)] \ effSurvive
Death, non−TB
[sum(cOutpt_DH;cHIV;cCXR;cOutpt_DH;cSputumXpert;cOutpt_DH)] \ effDeath
